# Perspective on the integration of radiomics and spatial omics in the analysis of the tumor microenvironment of bladder cancer and prospects for precision diagnosis and treatment

**DOI:** 10.3389/fimmu.2026.1821743

**Published:** 2026-07-08

**Authors:** Yan Dong, Yunwei Su

**Affiliations:** 1 Department of Radiology, The Fourth Affiliated Hospital of China Medical University, Shenyang, China; 2 Department of Urology, The Fourth Affiliated Hospital of China Medical University, Shenyang, China

**Keywords:** bladder cancer, immune microenvironment, radiomics, spatial omics, tumor heterogeneity

## Abstract

Bladder cancer, a common malignant tumor of the urinary system, possesses a highly heterogeneous tumor immune microenvironment that critically influences disease progression, treatment response, and patient prognosis. The limitations of traditional tissue biopsies have spurred interest in non-invasive, dynamic, and spatially resolved assessment strategies. Radiomics enables non-invasive quantification of macroscopic tumor phenotypes through high-throughput extraction of medical imaging features, while spatial omics technologies, such as single-cell and spatial transcriptomics, reveal the fine-grained spatial architecture of cellular and molecular components within the tumor microenvironment. This review systematically summarizes recent advances in radiomics and spatial omics for characterizing the bladder cancer immune microenvironment, with a focus on their synergistic applications in elucidating immune cell spatial distribution, stromal heterogeneity, immune checkpoint expression, and therapy response prediction. Furthermore, we critically examine the current challenges in multi-omics data integration, model generalization, and clinical translation, and outline future directions driven by artificial intelligence, including multimodal fusion, dynamic monitoring, and personalized therapeutic strategies. This review aims to provide a theoretical foundation and technical outlook for precision medicine in bladder cancer, facilitating the translation of imaging-spatial omics from basic research to clinical practice.

## 1 Introduction

Bladder cancer is one of the most common malignant tumors of the urinary system worldwide, and its high incidence and mortality rates pose a persistent disease burden on society. Epidemiological studies have shown that the occurrence of bladder cancer is associated with various risk factors, among which smoking is the primary pathogenic factor, with the incidence in smokers significantly higher than that in non-smokers (1). According to the depth of tumor invasion, bladder cancer can be divided into non-muscle-invasive bladder cancer (NMIBC) and muscle-invasive bladder cancer (MIBC). MIBC presents significant clinical challenges due to its aggressive nature, high recurrence rates, and propensity for metastasis. Current treatment options for bladder cancer include surgical resection, radiotherapy, chemotherapy, and the rapidly developing immunotherapy in recent years. Although existing therapies have extended the survival of patients to some extent, they still generally face issues such as limited efficacy, easy recurrence, and drug resistance, particularly in advanced or metastatic patients, where treatment effects urgently need improvement (2, 3) The differences in treatment responses largely stem from the high heterogeneity within tumors, especially the complex composition of the tumor microenvironment.

In recent years, the role of the tumor immune microenvironment in the occurrence, progression, and treatment response of bladder cancer has garnered increasing attention. The tumor immune microenvironment is a dynamic functional unit composed of various cellular components (including tumor cells, immune cells, fibroblasts, and stromal cells) and non-cellular components (such as the extracellular matrix). These components interact through complex interactions to regulate tumor immune evasion, angiogenesis, chemotherapy resistance, and metastatic ability (4–6). Research indicates that the immune microenvironment of bladder cancer exhibits high heterogeneity, where different immune cell subsets, such as CD8^+^ cytotoxic T cells, regulatory T cells, natural killer cells, and tumor-associated macrophages (TAMs), play critical roles in tumor progression and immune regulation (7, 8). Furthermore, the expression levels of immune checkpoint molecules such as PD-1/PD-L1 are closely related to the treatment response of bladder cancer patients to immune checkpoint inhibitors, becoming important targets for immunotherapy (9, 10). Although immunotherapy offers hope for some patients, the overall response rate remains unsatisfactory, and mechanisms of immune tolerance and immune evasion persist as current clinical challenges.

Therefore, a deep analysis of the cellular and molecular composition, spatial structure, and dynamic evolution of the bladder cancer immune microenvironment is not only helpful in revealing the intrinsic mechanisms of tumor biological behavior but also provides a theoretical basis for optimizing immunotherapy strategies and exploring new pathways for personalized treatment. Through systematic analysis of immune cell infiltration patterns, related gene expression, and immune regulatory networks, it is expected that more reliable biomarkers will be identified in the future, promoting the shift of bladder cancer treatment from traditional models to precise immune regulation.

The traditional assessment of the tumor immune microenvironment primarily relies on tissue biopsies; however, this method has inherent sampling biases, making it difficult to comprehensively capture the spatial heterogeneity and dynamic evolution of tumors, thus limiting a deeper understanding of tumor biological behavior and the development of precise treatment strategies (11, 12). Although conventional imaging techniques (such as CT, MRI, and ultrasound) hold a fundamental position in the diagnosis and staging of bladder cancer, they still have significant limitations: CT has insufficient sensitivity for detecting lymph nodes and distant metastases, and MRI still has shortcomings in identifying early lesions (13). Additionally, traditional imaging cannot accurately reflect the molecular characteristics and immune status of tumors, making it challenging to meet the demands of precision medicine. With the development of high-throughput technologies such as single-cell RNA sequencing (scRNA-seq) and spatial transcriptomics (ST), researchers can now analyze the complexity of the bladder cancer immune microenvironment at the cellular resolution level and reveal its tissue architecture from a spatial dimension (14, 15). For example, scRNA-seq studies have identified various functionally distinct immune cell subtypes in bladder cancer, whose distribution and activity status are closely related to patient responses to immune checkpoint inhibitor therapy (16, 17). Spatial omics technologies further reveal the spatial distribution patterns of cells and intercellular interactions *in situ*, providing key insights into the mechanisms by which tumor cells mediate immune evasion and invasion through the local microenvironment (18–20). Meanwhile, radiomics, as an innovative multidimensional data mining method, provides a new perspective for non-invasive assessment of tumor biological behavior and immune status by extracting quantitative features from medical images through high-throughput techniques (21). In bladder cancer research, CT-based radiomics models can non-invasively assess the tumor-stroma ratio and the degree of immune cell infiltration; whereas MRI radiomics models combined with RNA sequencing can predict CD8A expression levels and immune cell infiltration characteristics, which can be used for prognosis assessment and predicting responses to immunotherapy (22). The introduction of deep learning technologies has further enhanced the feature extraction and predictive performance of radiomics, providing strong support for the diagnosis and treatment management of bladder cancer (23, 24).

In summary, the high heterogeneity and complexity of the bladder cancer immune microenvironment are core factors influencing disease progression and treatment response. Traditional assessment methods can no longer meet the current demands for precise diagnosis and treatment, while emerging spatial omics and radiomics technologies provide powerful tools for comprehensively, dynamically, and non-invasively analyzing the tumor immune microenvironment from the microscopic spatial structure and macroscopic imaging phenotype dimensions. This review will focus on the latest research progress regarding the bladder cancer immune microenvironment, emphasizing the principles, applications, and value of radiomics and spatial omics technologies in revealing its heterogeneity, guiding immunotherapy, and prognostic assessment, while also discussing the challenges and future directions of multi-omics integration, aiming to provide theoretical support and practical guidance for precision immunotherapy in bladder cancer.

## 2 Immune microenvironment of bladder cancer

**Figure 1:**
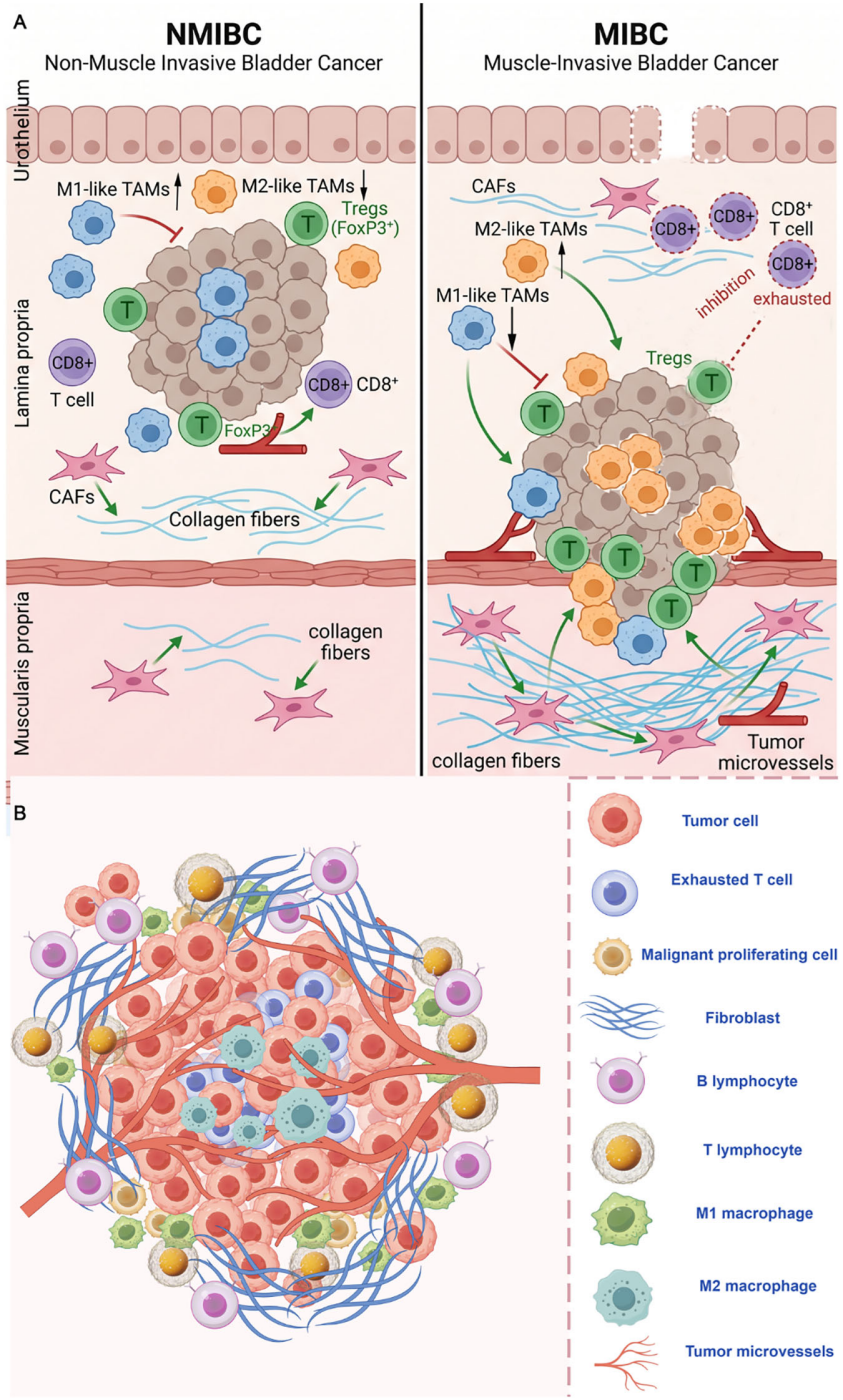
Spatial heterogeneity of the bladder cancer TIME. **(A)** Anatomical differences between NMIBC (left) and MIBC (right). NMIBC is confined to the urothelium and lamina propria, whereas MIBC invades through the muscularis propria, establishing the structural basis for TIME spatial heterogeneity. **(B)** Functional subregions of the MIBC TIME. The tumor core is characterized by an immunosuppressive landscape enriched with regulatory T cells (Tregs), M2-like TAMs, and cancer-associated fibroblasts (CAFs), which collectively drive CD8+ T cell exhaustion, with a paucity of pro-inflammatory M1-like TAMs. The invasive margin exhibits a higher density of cytotoxic CD8+ T cells and M1-like TAMs, indicative of a more immunoreactive milieu. The stromal compartment, composed of CAFs and a remodeled extracellular matrix (ECM), forms both a physical barrier and an immunosuppressive niche, further restricting immune cell infiltration and function.

The tumor immune microenvironment (TIME) of bladder cancer is a dynamic functional unit composed of various cellular and non-cellular components (such as tumor cells, immune cells, stromal cells, and extracellular matrix) that plays a decisive role in tumor occurrence, development, immune evasion, and therapeutic response (11). The spatial heterogeneity of this microenvironment, with its distinct cellular components and functional regions, is a key determinant of tumor behavior, as illustrated in **Figure 1**. Research indicates that the immune microenvironment of bladder cancer exhibits high heterogeneity, with different immune cell subpopulations such as CD8+ T cells, regulatory T cells (Tregs), natural killer cells (NK cells), and TAMs playing key roles in tumor progression and immune evasion (25, 26).

In-depth studies of the immune microenvironment in bladder cancer not only help to unveil the biological characteristics of the tumor but also provide a theoretical basis for optimizing immunotherapy regimens. Through systematic analysis of immune cell infiltration patterns, expression of immune-related genes, and immune regulatory pathways, potential immune therapeutic biomarkers can be identified to guide the formulation of individualized treatment strategies. For example, prognostic models constructed by integrating the immune cell composition and gene expression characteristics of the tumor microenvironment can effectively predict patient survival outcomes and responses to immunotherapy (27, 28). Furthermore, immune microenvironment classification based on m6A methylation modifications reveals the association between different immune phenotypes and treatment responses, providing new ideas for precision immunotherapy (29). These research findings emphasize the central role of the immune microenvironment in the treatment of bladder cancer, facilitating the shift from traditional therapies to immune regulation and personalized treatment.

### 2.1 Core cell components and their functions

The cell components of TIME mainly include adaptive immune cells, innate immune cells, and stromal cells, which collectively shape the immune status of tumors through complex interactions.

#### 2.1.1 Adaptive immune cells: T cells and B cells

In the immune microenvironment of bladder cancer, T cells and B cells serve as the core components of adaptive immunity, playing a decisive role in tumor occurrence, progression, and treatment response. CD8+ cytotoxic T lymphocytes (CTLs) are the main force in anti-tumor immunity; they directly kill tumor cells by recognizing antigens presented on the surface of tumor cells and releasing effector molecules such as perforin and granzyme. However, the bladder cancer microenvironment often inhibits CD8+ T cell function through various mechanisms, the most typical being the PD-1/PD-L1 immune checkpoint pathway, leading to T cell exhaustion and reduced cytotoxic efficacy. Studies have shown that bladder cancer patients with high PD-1/PD-L1 expression respond better to immune checkpoint inhibitor therapy, but the overall response rate remains limited, closely related to the immunosuppressive state in the tumor microenvironment (30). Additionally, heterogeneity of T cell subsets can be observed in bladder cancer tissues, including effector memory T cells and exhausted T cells, whose proportions and functional states are closely associated with patient prognosis (31). Notably, certain therapeutic approaches, such as cisplatin chemotherapy, can promote CD8+ T cell infiltration by activating the cyclic GMP-AMP synthase-interferon gene stimulator (cGAS-STING) pathway, enhancing anti-tumor immune effects, suggesting that regulating CD8+ T cell activity may become a key direction for improving the efficacy of bladder cancer immunotherapy (32).

In addition, regulatory T cells (Tregs) play a central role in the immunosuppression of bladder cancer. Tregs inhibit the proliferation and function of effector T cells by secreting immunosuppressive cytokines (such as IL-10, TGF-β) and expressing molecules such as cytotoxic T lymphocyte antigen 4 (CTLA-4) and PD-1, thereby promoting tumor immune evasion. Numerous studies have confirmed that high infiltration of Tregs in bladder cancer tissues is closely related to tumor progression, poor prognosis, and resistance to immunotherapy (33) In the immunosuppressive microenvironment, there is a positive feedback regulation between Tregs and other immunosuppressive cells such as TAMs, further exacerbating immune evasion (34). Interestingly, chemotherapy such as neoadjuvant cisplatin treatment can significantly reduce the number of Tregs, improve the tumor microenvironment, and enhance anti-tumor immune responses, a phenomenon particularly pronounced in patients with pathological complete response (7) Therefore, targeting Tregs or their regulatory pathways is expected to become an important strategy for reversing immunosuppression and enhancing the efficacy of immunotherapy.

The role of B cells in the immune microenvironment of bladder cancer is dualistic (35). On one hand, B cells can participate in anti-tumor immune responses through mechanisms such as producing antibodies, promoting antigen presentation, and activating T cells. Especially in muscle-invasive bladder cancer, the presence of tumor-infiltrating B cells and tertiary lymphoid structures (TLSs) is associated with better prognosis and stronger anti-tumor immune responses (35, 36). B cells within TLSs can differentiate into plasma cells, produce specific antibodies, enhance the phagocytic capacity of macrophages, and cooperate with T cells through the CXCL13-CXCR5 axis to promote anti-tumor immunity. On the other hand, certain B cell subpopulations, such as regulatory B cells (Bregs) and atypical B cells (ABCs), can secrete immunosuppressive factors like IL-10, inhibiting T cell activity and promoting tumor progression and resistance to immunotherapy (37, 38). In patients unresponsive to Bacillus Calmette-Guérin (BCG) immunotherapy, B cell-dominated TLSs are often enriched in the subepithelial region, accompanied by increased expression of immune exhaustion-related proteins, indicating the complexity of B cell immunoregulatory functions. Notably, B cell-related gene features (such as a high IgG1/IgA ratio) can serve as independent predictive factors for immunotherapy response and prognosis, and combining T cell characteristics can further enhance the accuracy of efficacy predictions (39).

Overall, T cells and B cells collaboratively regulate anti-tumor and immunosuppressive processes in the immune microenvironment of bladder cancer, with their subpopulation ratios, functional states, and spatial distributions determining the strength of tumor immune responses and clinical treatment outcomes. In the future, precisely regulating T cell activity, inhibiting immunosuppressive subpopulations such as Tregs and Bregs, and promoting the formation of beneficial B cell subtypes and TLSs may provide new breakthroughs for personalized immunotherapy in bladder cancer.

#### 2.1.2 Innate immune cells: macrophages, dendritic cells, and neutrophils

In the immune microenvironment of bladder cancer, innate immune cells include TAMs, Dendritic Cells (DCs), and Neutrophils, which play a crucial role in tumor occurrence, development, and response to immunotherapy. TAMs are typically classified into M1 and M2 types based on their function and phenotype. M1 macrophages exhibit pro-inflammatory and anti-tumor effects, capable of producing a large amount of inflammatory factors (such as IL-1β, TNF-α, inducible nitric oxide synthase, iNOS), promoting tumor cell apoptosis and inhibiting tumor growth (40). In contrast, M2 macrophages display immunosuppressive and pro-tumor characteristics, secreting factors such as IL-10 and TGF-β, which promote tumor cell proliferation, metastasis, and angiogenesis, and are closely associated with poor prognosis (41, 42). Studies have shown that bladder cancer cells can induce macrophage polarization towards the M2 type by secreting chemokines (such as CXCL12, CCL20, etc.) or releasing exosomes, further establishing an immunosuppressive microenvironment that promotes tumor progression (43–45). Additionally, certain signaling pathways, such as the phosphatidylinositol 3-kinase/protein kinase B (PI3K/Akt), signal transducer and activator of transcription 3/6 (STAT3/6), and Parkin (PRKN)-mediated mitophagy, play a critical role in the M2 polarization process (41, 46). Notably, molecules that regulate TAM polarization (such as Claudin 6, CLDN6; insulin-like growth factor 2 mRNA-binding protein 2, IGF2BP2; long non-coding RNA LINC01140, etc.) have been confirmed to be closely related to the prognosis of bladder cancer and the response to immunotherapy (47–49). In therapeutic terms, BCG immunotherapy enhances anti-tumor immune responses by activating TAMs to polarize towards the M1 type, and its efficacy is closely related to the ratio of M1/M2 macrophages in the tumor microenvironment (50, 51).These findings suggest that interventions targeting TAM polarization may become a new strategy for immunotherapy in bladder cancer.

Dendritic cells (DCs), as the most important antigen-presenting cells, are responsible for capturing, processing, and presenting tumor-associated antigens to T cells in the immune microenvironment of bladder cancer, thereby activating tumor-specific T cell immune responses. Studies have shown that the quantity and maturation status of DCs in the tumor tissues and peripheral blood of bladder cancer patients are closely related to the response to immunotherapy (52, 53). High-dose radiotherapy and BCG treatment can induce immunogenic cell death (ICD) in tumor cells, releasing damage-associated molecular patterns (DAMPs), promoting the recruitment and maturation of DCs, enhancing their antigen presentation capabilities, and activating CD8+ cytotoxic T cells (54, 55). Furthermore, the functional status of DCs can be influenced by immunosuppressive factors in the tumor microenvironment, such as the chemokine CXCL9 secreted by tumor-associated DCs, which can upregulate PD-L1 expression in tumor cells and inhibit T cell activity (56). Immunotherapeutic strategies targeting DCs (such as DC vaccines and CD40 agonists) have shown good anti-tumor effects in preclinical and clinical studies of bladder cancer by enhancing the antigen presentation and T cell activation capabilities of DCs, thereby improving the immunotherapy response rate in patients (52, 57, 58). Therefore, optimizing the antigen presentation function and maturation status of DCs or blocking their immunosuppressive signals is expected to further enhance the efficacy of immunotherapy for bladder cancer.

Neutrophils, as important innate immune cells in the microenvironment of bladder cancer, exhibit a dual effect of pro-inflammatory and immunosuppressive responses. Tumor-Associated Neutrophils (TANs) can promote the proliferation, migration, angiogenesis, and lymphangiogenesis of tumor cells by releasing Reactive Oxygen Species (ROS), chemokines (such as IL-8/CXCL8), Hepatocyte Growth Factor (HGF), and others (59, 60). IL-8 secreted by bladder cancer cells enhances the recruitment and activation of neutrophils, while HGF secreted by neutrophils can upregulate the expression of IL-8 in tumor cells through c-Fos, forming a positive feedback loop that further amplifies the pro-tumor effects (59). Additionally, neutrophils can form Neutrophil Extracellular Traps (NETs), which endow tumor cells with resistance to radiotherapy and chemotherapy, and high levels of NETs are closely associated with poor chemotherapy response and adverse prognosis in bladder cancer patients (61, 62). However, in early bladder cancer, neutrophils can also exert a certain protective effect by producing ROS and participating in anti-tumor immune responses (63). It is noteworthy that the distribution of TANs varies among different molecular subtypes of bladder cancer, and their functions are regulated by multiple factors such as the tumor microenvironment, gender, and gut microbiota (64, 65). These evidences suggest that the functional plasticity of TANs and their interactions with factors like IL-8 and HGF determine their dual roles in bladder cancer progression and immunotherapy.

Overall, macrophages, dendritic cells, and neutrophils interact through complex signaling networks in the immune microenvironment of bladder cancer, collectively shaping the immune status of the tumor and influencing tumor incidence, progression, and response to immunotherapy. A deeper understanding of the polarization, functional states of these innate immune cells, and their interaction mechanisms with key factors will provide a theoretical basis and new targets for precise immune intervention in bladder cancer.

#### 2.1.3 Fibroblast and extracellular matrix remodeling

Fibroblasts, particularly cancer-associated fibroblasts (CAFs), play a central role in the TIME of various solid tumors, including bladder cancer. CAFs promote tumor growth, invasion, and metastasis by secreting various cytokines (such as TGF-β1, FGF2, CXCL14, etc.) and remodeling the extracellular matrix (ECM) (66–68). In bladder cancer, specific CAF subtypes, such as inflammatory CAFs (iCAFs), form complex signaling networks with tumor stem cells and immune cells via FGF2 and CCL2, maintaining tumor stemness and epithelial-mesenchymal transition (EMT) (69). CXCL14 secreted by CAFs can activate CCR7 on the surface of tumor cells, thereby initiating the STAT3 signaling pathway, enhancing DNA repair capabilities, and promoting drug resistance, a mechanism that has been validated in clinical samples and in vitro and in vivo models (68). Additionally, CAFs can activate the NOTCH2/HEY1 pathway through molecules like MFAP5, further stimulating the proliferation and metastasis of tumor cells (70).

In bladder cancer, specific CAF subtypes, such as inflammatory CAFs (iCAFs), form complex signaling networks with tumor stem cells and immune cells via FGF2 and CCL2, maintaining tumor stemness and epithelial-mesenchymal transition (EMT). The main components of the ECM include collagen, hyaluronic acid, and fibronectin; the deposition and rearrangement of these molecules not only change the stiffness and structure of the tissue but also provide a “track” for the movement and invasion of tumor cells (71, 72). In the TIME of bladder cancer, CAFs promote ECM remodeling by upregulating genes such as FAP, CALD1, and VCAN, forming a dense matrix barrier that hinders immune cell (such as CD8+ T cells) infiltration into the tumor core, thereby inducing immune rejection and tumor immune escape (73, 74). Notably, the stiffness and arrangement of the ECM can dynamically regulate the migration capacity and drug resistance of tumor cells, and the spatial structural heterogeneity of CAF-ECM is an important material basis for tumor heterogeneity (75).

In summary, fibroblasts and their mediated extracellular matrix remodeling serve as a structural backbone of the bladder cancer TIME. CAFs promote tumor growth, invasion, metastasis, and drug resistance by secreting various cytokines and remodeling the ECM, forming a complex interaction network with immune cells, vascular endothelial cells, and significantly influencing the biological behavior and therapeutic response of tumors. In the future, in-depth analysis of the spatial structure and functional heterogeneity of CAF-ECM will provide important references for the precise diagnosis and treatment of bladder cancer and the development of new targeted strategies.

### 2.2 Dynamic regulation and spatial heterogeneity of the bladder cancer immune microenvironment

The bladder cancer immune microenvironment is a highly dynamic and spatially heterogeneous complex ecosystem, characterized primarily by the establishment of immune escape mechanisms, T cell exhaustion, and co-evolution between the tumor and the immune system (76, 77). First, the aberrant activation and regulatory networks of immune checkpoint molecules are key drivers of immune escape, involving the synergistic effects of PD-1/PD-L1, CTLA-4, and emerging checkpoints such as VISTA and TIM-3 (78–80). Second, persistent antigen stimulation and remodeling of the metabolic microenvironment (e.g., lactate accumulation, tryptophan depletion) lead to a progressive state of T cell exhaustion, manifested by the loss of effector function and co-expression of inhibitory receptors (81, 82). Finally, the continuous spatial interplay between the tumor and the immune system results in distinct spatial architectures, including immune-inflamed, excluded, and desert phenotypes; this spatial heterogeneity directly influences therapeutic responses (83). Understanding these intertwined mechanisms holds significant clinical implications for developing multi-targeted combination immunotherapy strategies.

#### 2.2.1 Mechanisms of immune evasion and regulation by immune checkpoint molecules

Immune evasion in bladder cancer is a multi-layered, systematic process. Its core lies in the TIME synergistically dismantling host immune surveillance through three pillars: aberrant activation of immune checkpoint axes, antigen presentation defects, and inhibitory cells coupled with metabolic remodeling (31, 84). Among these, immune checkpoint molecules such as PD-1/PD-L1 and CTLA-4 play pivotal roles 11. High expression of PD-L1 on tumor cells and tumor-associated immune cells inhibits T cell activation and proliferation by binding to PD-1 on the surface of T cells, thereby helping tumor cells evade immune surveillance (85). Its expression is precisely regulated by multiple signaling pathways; for instance, Fusobacterium nucleatum can upregulate PD-L1 expression by activating the IL22/STAT3 signaling cascade, thus promoting bladder cancer progression and immune evasion (86). The androgen receptor (AR) can directly bind to androgen response elements in the PD-L1 promoter region to transcriptionally repress PD-L1 expression; therefore, AR loss or low expression may lead to PD-L1 upregulation, further promoting immune evasion (87). Similarly, the transcription factor FOXP3 can bind to the PD-L1 promoter to promote its expression, synergizing with interferon-γ to induce an immunosuppressive environment (88). Furthermore, CTLA-4 is highly expressed on regulatory T cells (Tregs) and activated T cells; by competitively binding to CD80/CD86 on the surface of antigen-presenting cells (APCs), it inhibits co-stimulatory signals, thereby weakening T cell priming and activation (85). Meanwhile, emerging checkpoints such as TIM-3, LAG-3, TIGIT, and VISTA cooperate with PD-1 to form a multi-layered inhibitory network that exacerbates T cell exhaustion (85, 89). Secondly, tumor cells create obstacles in antigen presentation and recognition by downregulating MHC class I molecules, disrupting antigen processing transporters (such as TAP), and expressing non-classical HLA-E/G molecules, rendering cytotoxic T lymphocytes unable to effectively recognize and attack tumors (90–92). Concurrently, the massive infiltration of regulatory T cells, myeloid-derived suppressor cells, and M2-type TAMs in the TIME comprehensively suppresses effector immune cell function by secreting inhibitory factors such as IL-10 and TGF-β, while consuming arginine and producing reactive oxygen species (93–95). Moreover, metabolic abnormalities such as hypoxia, acidosis, and lactate accumulation further impair immune cell adaptability through pathways involving HIF-1α (96). The interplay of these cellular and molecular mechanisms collectively shapes the immunosuppressive microenvironment of bladder cancer.

#### 2.2.2 T cell exhaustion status and functional remodeling

T cell exhaustion is a key feature of the immune microenvironment in bladder cancer. Within the bladder cancer microenvironment, T cell exhaustion is a highly dynamic and multi-layered process encompassing early dysfunction, terminal phenotype locking, and spatially heterogeneous distribution (31, 97). Early exhaustion is primarily driven by persistent tumor antigen stimulation and co-inhibitory signals such as PD-1/PD-L1, resulting in partial loss of T cell effector functions (e.g., secretion of IFN-γ, TNF-α, and granzyme B) while retaining proliferative capacity. At this stage, cells upregulate PD-1 and TIM-3 while maintaining TCF1 and TOX expression, thereby preserving stem-like self-renewal properties and functional reversibility (98), and retaining potential responsiveness to immune checkpoint blockade therapy. However, accompanied by metabolic reprogramming shifting from oxidative phosphorylation to glycolysis, mitochondrial dysfunction and enhanced fatty acid oxidation further exacerbate functional decline (99). As exhaustion deepens, terminally exhausted T cells co-express multiple inhibitory receptors including PD-1, TIM-3, LAG-3, TIGIT, and CD39, with nearly complete loss of effector function and proliferative capacity; within the transcription factor network, TOX and the NR4A family are significantly upregulated, whereas TCF1 and EOMES are downregulated, and an irreversible lock occurs at the epigenetic level—effector gene loci (such as IFNG and GZMB) are sealed by repressive histone modifications, while inhibitory receptor gene loci (such as PDCD1 and HAVCR2) remain open (100–102). Single-cell and spatial transcriptomics have further revealed significant heterogeneity in T cell exhaustion, identifying various subsets including precursor, intermediate, and terminal stages. Exhausted T cells are mainly enriched in the tumor core and invasive front, forming immunosuppressive niches with tumor and stromal cells. Notably, B cells and dendritic cells within tertiary lymphoid structures (TLS) can partially reverse T cell exhaustion through potent antigen-presenting capabilities, offering new avenues for improving immunotherapeutic efficacy (103, 104).

#### 2.2.3 Tumor-immune co-evolution and spatial heterogeneity

A dynamic co-evolutionary relationship exists between bladder cancer and the immune system, a process that profoundly shapes disease progression and therapeutic response (45, 93). Selective pressure exerted by the immune system drives clonal evolution of the tumor; through “elimination-equilibrium-escape” immunoediting, clones with immune evasion phenotypes (such as MHC-I loss and PD-L1 upregulation) are selected. This is accompanied by increased tumor mutational burden (TMB) and genomic instability, leading to exacerbated intratumoral heterogeneity and resistance to immunotherapy (91, 105). Concurrently, tumor cells actively remodel the microenvironment by secreting chemokines to recruit suppressive cells such as myeloid-derived suppressor cells (MDSCs) and regulatory T cells (Tregs), and by utilizing exosomes to deliver immunomodulatory molecules, thereby converting the microenvironment into a state of sustained suppression (86, 106). Remodeling of the extracellular matrix (ECM) further establishes physical barriers that restrict the infiltration of cytotoxic T cells (107). These co-evolutionary dynamics offer critical insights for therapeutic strategies: early intervention, combination targeting of multiple immune checkpoints (e.g., PD-1/CTLA-4), and dynamic monitoring coupled with personalized treatment based on liquid biopsies (e.g., ctDNA) represent key directions for overcoming resistance and enhancing efficacy (108, 109). For instance, single-cell and spatial omics technologies have revealed that the distribution of immune cells in bladder cancer tissues is not uniform, exhibiting distinct spatial architectures such as immune-inflamed, immune-excluded, and ‘immune-desert’ types (109, 110). In immune-inflamed types, there is a high abundance of anti-tumor immune cells such as CD8^+^ T cells, natural killer cells, and dendritic cells, along with upregulated expression of immune checkpoint molecules; whereas in immune-desert types, suppressive cells such as M2 macrophages and fibroblasts predominate (111, 112). This spatial heterogeneity directly impacts therapeutic outcomes; the distance between immune and tumor cells, their distribution density, and aggregation status collectively determine the activation level of the immune microenvironment and the response to treatment (113, 114). Furthermore, the formation and functional status of tertiary lymphoid structures serve as a spatial manifestation of tumor-immune interactions and are associated with better prognosis and responses to immunotherapy (115, 116). Through metabolic reprogramming (such as enhanced glycolytic activity), tumor cells not only support their own growth but also exhibit metabolic features linked to specific immune infiltration patterns; for example, high expression of glycolysis-related genes correlates with CD8^+^ T cell functional exhaustion and PD-L1 upregulation, predicting poorer responses to immunotherapy (117, 118). This dynamic interplay, wherein tumor cells adapt and remodel the microenvironment by altering their metabolism and signaling pathways under immune selective pressure while the immune system correspondingly adjusts its response, lies at the core of understanding bladder cancer progression and therapeutic resistance (86, 119, 120).

### 2.3 Implications for precision immunotherapy

A deep understanding of the aforementioned intertwined mechanisms is crucial for developing more effective immunotherapeutic strategies for bladder cancer. Targeting the complex immune checkpoint network, combined blockade of different pathways (such as PD-1/PD-L1 alongside other emerging checkpoints) may prove more effective than monotherapy (85, 105, 121). Reversing T cell exhaustion requires a multi-pronged approach, including blocking inhibitory signals (e.g., PD-1/PD-L1), improving the metabolic microenvironment (e.g., targeting lactate metabolism or IDO1), and modulating immunosuppressive cells (e.g., targeting Tregs or M2 macrophages) (122, 123). Given the high spatial heterogeneity of the TIME, future treatments will need to incorporate information from spatial omics to formulate personalized strategies. For instance, for ‘immune-desert’ tumors, it may be necessary to first employ methods (such as radiotherapy or certain chemotherapeutic agents) to convert ‘cold tumors’ into ‘hot tumors’ before combining with immune checkpoint inhibitors (124–126). Meanwhile, leveraging non-invasive techniques like radiomics to assess overall tumor heterogeneity and immune status, combined with microscopic cellular geographical information revealed by spatial omics, will offer the potential to achieve truly dynamic precision therap (127–129).

In summary, the immune microenvironment of bladder cancer is a dynamic ecosystem characterized by high spatial heterogeneity, composed of various immune cells and stromal cells interacting through complex molecular networks. Its composition and state directly determine the tumor’s invasiveness, metastatic potential, and response to treatment (especially immunotherapy). A deeper analysis of the spatial organizational characteristics of the tumor center and edge, TLS, and other immune cell subpopulations can help discover new prognostic and treatment response biomarkers, providing a theoretical basis for formulating precise immunotherapy strategies. Currently, the “gold standard” assessment of the TIME still relies on tissue biopsy. However, this method has unavoidable limitations: first, it is an invasive procedure that can cause trauma and potential risks to patients; second, due to the significant spatial heterogeneity of tumors, single-point biopsies are difficult to reflect the overall picture of the tumor, easily leading to sampling errors and misjudgments; finally, biopsies cannot provide real-time and repeatable monitoring of dynamic changes in the TIME. These limitations severely restrict the practice of precision medicine in the field of bladder cancer. Therefore, developing new techniques and strategies that can non-invasively, comprehensively, and dynamically assess the TIME has become an urgent need in current clinical practice.

### 2.4 Technological innovation: radiomics and spatial omics as a new paradigm for decoding the complexity of the TIME

In summary, the immune microenvironment of bladder cancer is a highly organized and dynamically changing complex ecosystem. At its core is a tumor cell-centered environment surrounded by infiltrating immune cells (including both effector and suppressive types) and activated stromal cells (such as CAFs). All these cells are immersed in a non-cellular network composed of extracellular matrix, soluble factors, and metabolic products. These components interact through a complex signaling network, collectively determining the overall immune functional status of the TIME—whether it tends toward immune clearance or progresses to immune escape and treatment resistance. Therefore, systematically analyzing the composition and interaction relationships of the TIME has become the cornerstone for developing effective immunotherapy strategies, and this is precisely the broad stage on which emerging technologies such as radiomics and spatial omics can play their revolutionary roles.

Radiomics, as a high-throughput quantitative analysis technology, can extract massive amounts of feature information from conventional medical images (such as CT, MRI, PET) that are not recognizable to the human eye, thereby non-invasively decoding tumor heterogeneity, characterizing its microenvironment status, and demonstrating tremendous potential in diagnosis, prognosis prediction, and treatment response evaluation. On the other hand, breakthroughs in spatial omics technologies, particularly spatial transcriptomics and spatial proteomics, enable us to map the “cellular landscape” of the TIME at the molecular level while preserving the original spatial structure of the tissue, accurately revealing the spatial localization, functional states, and interaction networks of different cell populations. Radiomics and spatial omics provide complementary perspectives from the dimensions of “macro imaging phenotypes” and “micro molecular spaces,” respectively. The integration of the two is expected to achieve a leap from “seeing” the tumor to “understanding” the tumor, providing an unprecedented integrative analytical framework for comprehensively deciphering the complexity of the bladder cancer TIME.

Therefore, this article aims to systematically review the application progress and integration prospects of radiomics and spatial omics in the study of the TIME in bladder cancer. We will first outline the technical processes of radiomics and spatial omics separately and their latest discoveries in revealing the immune microenvironment, stromal heterogeneity, and treatment responses in bladder cancer; further, we will focus on discussing the methods, application examples, and current challenges of their integrated analysis; finally, we will look ahead to the clinical translational potential and future development directions of this integration strategy in promoting personalized precision diagnosis and treatment of bladder cancer.

## 3 Radiomics: decoding the macro view of bladder cancer TIME

Tissue biopsy is the gold standard for assessing the TIME, but its invasive nature and inherent sampling bias make it difficult to comprehensively characterize the highly heterogeneous spatiotemporal panorama of bladder cancer. Traditional imaging techniques (such as CT and MRI) play a fundamental role in the diagnosis and clinical staging of bladder cancer, but they have certain limitations. For example, CT has limited sensitivity in detecting lymph nodes and distant metastases, and although MRI can better differentiate between muscle-invasive and non-muscle-invasive disease, it still has shortcomings in identifying early lesions (13). Furthermore, traditional imaging fails to accurately reflect the molecular characteristics and immune status of tumors, which restricts the implementation of precision therapies. With the development of imaging technology, molecular imaging techniques such as PET/CT and PET/MRI have gradually been applied in bladder cancer, providing new approaches for non-invasive assessment of the tumor immune microenvironment (130). Additionally, traditional imaging diagnosis primarily relies on the subjective experience and visual assessment of doctors, making it difficult to quantify the complex structures within tumors, let alone directly reveal the underlying molecular and immune states (131, 132). This limitation has spurred the demand for technologies capable of non-invasively and comprehensively assessing the entire tumor ecosystem. In this context, radiomics has emerged, which transforms medical imaging into a mineable data field by high-throughput extraction and analysis of a large number of high-dimensional features from multimodal images (such as CT, PET, and MRI), and combines artificial intelligence technologies, especially machine learning and deep learning, to achieve precise segmentation and feature analysis of tumor subregions, greatly enhancing the interpretative capacity regarding tumor biological behavior (133, 134). Its core value lies in the ability to non-invasively and repetitively quantify the entire tumor and its macro environment, indirectly capturing the internal structural heterogeneity by reflecting differences in density, texture, and physiological characteristics across different regions of the tumor. This macro heterogeneity phenotype is a concentrated representation of complex underlying microscopic cellular and molecular activities in imaging, thus providing a unique “macro view” for indirectly inferring the overall state of bladder cancer TIME. Therefore, in bladder cancer research, CT-based “habitat” radiomics and deep learning models can non-invasively assess tumor stromal heterogeneity, predict recurrence-free survival, and immune therapy response, reflecting the significant value of radiomics in the assessment of the tumor immune microenvironment (21).

### 3.1 Types and extraction methods of radiomics features

Radiomics features primarily include multidimensional quantitative information such as morphological features, texture features, and gray-level co-occurrence matrices. These features can be extracted in high throughput from medical images to assist in tumor typing, grading, and prognosis assessment. Morphological features typically describe parameters such as the geometric shape, volume, and boundary clarity of tumors, reflecting their growth patterns and invasiveness. For instance, MRI radiomics, as a quantitative imaging biomarker, has demonstrated potential in bladder cancer staging, grading, and prognosis prediction (135). Texture features reveal intratumoral heterogeneity and microenvironmental changes by analyzing the spatial heterogeneity of pixel intensity distributions in images. Commonly used texture features include the Gray-Level Co-occurrence Matrix (GLCM) and the Gray-Level Size Zone Matrix (GLSZM) (136). Among these, GLCM can reflect the complexity and regularity within tumor tissues and has been confirmed to be closely related to the grading and typing of bladder cancer.

The extraction process for radiomics features typically includes steps such as image preprocessing, tumor segmentation, feature extraction, and feature selection. Image preprocessing involves operations like denoising, normalization, and resampling to ensure feature stability and reproducibility. Tumor segmentation can be performed either manually or using automatic or semi-automatic segmentation algorithms; in recent years, deep learning methods such as Convolutional Neural Networks (CNN) have significantly improved segmentation accuracy and efficiency in automatic applications (24). During the feature extraction phase, hundreds to thousands of features can be batch-extracted from Regions of Interest (ROI) using open-source tools such as PyRadiomics. Feature selection often employs statistical methods like minimum Redundancy Maximum Relevance (mRMR) and LASSO to screen for features most relevant to clinical outcomes, thereby reducing redundancy and the risk of overfitting (137).

Machine learning and deep learning play a core role in the extraction and analysis of radiomics features. Traditional machine learning methods, such as Random Forest (RF), can construct classification and prediction models based on handcrafted features to achieve discrimination of bladder cancer staging (138). Deep learning technologies, particularly CNN-based automatic feature extraction, not only learn high-order, abstract features from raw images but also integrate with traditional radiomics features to enhance model generalization and prediction accuracy. For example, deep residual networks can automatically identify complex imaging features associated with tumor grading and staging (139). It is worth noting that deep learning models rely heavily on large sample sizes and multi-center data, and their external validation and generalization capabilities still require further strengthening.

In bladder cancer radiomics research, the continuous enrichment and optimization of feature types and extraction methods provide a solid foundation for achieving precise tumor typing and non-invasive assessment of the immune microenvironment. In the future, with the continued advancement of artificial intelligence algorithms and the integration of multimodal imaging data, the automation and standardization of radiomics feature extraction, along with their deep fusion with molecular omics data, are expected to further promote the development of personalized diagnosis and treatment.

### 3.2 Preprocessing and standardization of radiomics data

Preprocessing and standardization of radiomics data are critical links ensuring subsequent feature extraction, model training, and result reproducibility. First, image segmentation, as the foundation of the entire workflow, directly affects the accuracy and biological relevance of subsequent features. Recent mainstream studies have mostly adopted 3D Slicer and PyRadiomics for manual feature extraction (140). The accuracy of the segmented region is crucial for feature extraction; different segmentation methods can lead to significant differences in feature values, a phenomenon that is particularly prominent in multi-center studies of tumors such as bladder cancer. Therefore, adopting standardized segmentation workflows and multi-reader consensus helps improve data comparability and the robustness of subsequent models.

Resampling technology is an indispensable step in the preprocessing workflow, mainly used to adjust imaging data obtained from different scanners and under different parameters to ensure consistent spatial resolution. Common methods include resampling all voxels to a unified spatial resolution (e.g., 1×1×1 mm^3^) to eliminate feature shifts caused by differences in scanning parameters (141). Additionally, some studies have introduced AI-driven high-resolution resampling algorithms (such as SMORE), which can enhance model predictive capability while preserving image details (142). Resampling not only improves feature reproducibility but also provides technical guarantees for the integrated analysis of multi-center and heterogeneous data. In practical applications, the selection of resampling parameters must balance image quality, computational efficiency, and the stability of subsequent features.

Normalization techniques primarily address image intensity values (or signal intensities) to eliminate signal differences arising from different equipment, scanning protocols, and patients. Mainstream normalization methods include those based on whole-image mean, reference tissues (such as urine), and histogram matching (143). Studies have found that normalization and magnetic field inhomogeneity correction are crucial for enhancing the ability of MRI radiomics features to distinguish tumor tissue types (141). It is worth noting that different normalization methods have varying impacts on feature stability; some methods may significantly affect high-order texture features. Therefore, in multi-center studies, the standardization and transparent reporting of normalization schemes are particularly critical.

Data quality control permeates every stage of radiomics and is a prerequisite for ensuring research reliability and clinical translation value. Quality control measures include preprocessing parameters such as voxel resampling, normalization, and discretization to improve feature stability and reproducibility (141). For example, N4ITK bias field correction was used in this study for magnetic field inhomogeneity correction of MRI data, significantly improving feature discriminative ability (144). Furthermore, for multi-center data, batch effect correction algorithms such as ComBat can reduce systematic differences between different devices and centers, but their effectiveness depends on specific MRI types, feature categories, and tasks, and they have certain limitations (145). Achieving high-quality data control also relies on standardized image acquisition, segmentation, and feature extraction workflows, as well as strict process documentation and collaboration among multi-centers.

## 4 Spatial genomics: mapping the microenvironment of the Tumor Immune Microenvironment

Traditional methods, such as immunohistochemistry, flow cytometry, and even the emerging single-cell RNA sequencing (scRNA-seq), have greatly enhanced our understanding of the cellular composition of the tumor immune microenvironment (TIME) in bladder cancer. scRNA-seq, in particular, reveals the heterogeneity of immune cell subpopulations within tumors and their potential functional states with unprecedented resolution (75). However, these techniques inherently dissociate tissues, resulting in the loss of crucial spatial contextual information. This prevents us from knowing the precise localization of specific cell subpopulations within the tissue, their interactions with neighboring cells, and how these spatial features collectively shape local immune activation or suppression “niches.” This “spatial blind spot” severely limits our in-depth understanding of the functional heterogeneity of TIME; for example, it cannot explain why the same proportion of immune cells can produce vastly different anti-tumor immune effects in different patients or in different regions of the same tumor.

The rise of spatial genomics technology perfectly compensates for this critical deficiency. Spatial transcriptomics and spatial proteomics, as core technologies in this field, allow for high-throughput, high-resolution *in situ* analysis of gene expression and protein distribution while fully preserving the original spatial structure of the tissue (146, 147). They provide researchers with a “molecular microscope,” capable not only of answering “what” cells and molecules are present in TIME but also of precisely revealing “where” they are located and “how” they interact. This section will systematically review how these technologies enable us to draw an unprecedented micro-map of the bladder cancer TIME, rich in spatial coordinates, and explain how this map deepens our understanding of immune regulatory mechanisms and advances the development of precision therapeutic strategies.

### 4.1 Technical introduction: principles and platforms of spatial transcriptomics and spatial proteomics

#### 4.1.1 Spatial transcriptomics technology

Spatial transcriptomics technology is a cutting-edge method that combines RNA sequencing with spatial localization information, enabling the simultaneous acquisition of transcriptomic expression data and the spatial distribution of cells within tissue sections. The core principle of this technology lies in the use of spatially encoded capture probes or microarrays to capture mRNA from specific locations in tissue sections and perform high-throughput sequencing, thus achieving spatial visualization of the entire transcriptome expression profile. This method not only preserves the spatial structure of the tissue but also reveals the spatial heterogeneity of cell types, states, and their interactions within the tumor microenvironment, greatly enriching our understanding of tumor biology (15).

Currently, various representative technological platforms have emerged in the field of spatial transcriptomics, each with its own resolution and applicable scenarios. Visium Spatial Gene Expression (10x Genomics) is one of the most widely used platforms, with a spatial resolution of up to 55μm, capable of large-area, high-throughput spatial transcriptomic analysis of tissue sections. Additionally, the Digital Spatial Profiler (NanoString GeoMx) combines immunofluorescence imaging with high-throughput sequencing, supporting highly flexible spatial profiling of regions of interest (ROI). Higher resolution platforms such as MERFISH, seqFISH, and Stereo-seq can achieve single-cell level spatial transcriptomic measurements, providing a technological basis for elucidating fine interactions between cells in the tumor microenvironment (148). Different platforms have their advantages in resolution, throughput, and tissue applicability, allowing researchers to choose the appropriate technology based on actual needs.

The advantages of spatial transcriptomics technology are mainly reflected in its ability to reveal the diversity of cell types and their spatial distribution within tumor tissues while preserving the spatial structure of the tissue. This technology holds unique value, especially in the study of tumor immune microenvironments. For example, studies have found that spatial transcriptomic analysis can identify the spatial mixing characteristics of tumors and immune cells, revealing tumor heterogeneity and patterns of immune cell infiltration (149, 150). Furthermore, spatial transcriptomics can be integrated with multi-omics data such as single-cell RNA sequencing and spatial proteomics, further enhancing the ability to analyze the complexity of the tumor microenvironment. Notably, spatial transcriptomics technology can uncover spatial characteristics related to intercellular communication, molecular pathway activation, and treatment resistance when analyzing immune microenvironments of highly heterogeneous tumors such as bladder cancer, thus providing molecular bases for the formulation of personalized treatment strategies (151).

Despite the numerous advantages of spatial transcriptomics technology, there are certain limitations. Firstly, current mainstream technologies face a trade-off between spatial resolution and detection throughput, with some platforms struggling to achieve whole transcriptome analysis at the single-cell level. Secondly, the preparation, preservation, and processing of tissue samples have high demands for retaining spatial information and data quality, making the technical operation complex and costly. Additionally, the application of spatial transcriptomics in diseases such as bladder cancer has enhanced the understanding of tumor heterogeneity and microenvironments, and it holds promise for future personalized treatment (15). In practical applications, effectively connecting spatial information with functional validation and clinical translation is also a pressing issue that needs to be addressed in this field.

Overall, as a powerful tool for analyzing the spatial structure and functional heterogeneity of the immune microenvironment in bladder cancer, spatial transcriptomics technology is driving tumor spatial biology toward higher precision and deeper insights. With continuous improvements in technical resolution, throughput, and data analysis capabilities, spatial transcriptomics is expected to play a greater role in tumor molecular typing, immune therapy target discovery, and precision medicine.

#### 4.1.2 Spatial proteomics technology

Spatial proteomics technology is a cutting-edge tool in the field of tumor microenvironment research in recent years, capable of obtaining spatial distribution information of protein expression simultaneously on tissue sections. Multiplex immunofluorescence (mIF) and mass spectrometry imaging (MSI) are the two core technologies. mIF detects multiple proteins simultaneously on a single tissue section using various fluorescently labeled antibodies, combined with high-resolution imaging and image analysis, allowing for the fine recognition of different cell types and their functional states in tumors and their immune microenvironments. MSI directly detects the spatial distribution of proteins, peptides, or metabolites on tissue sections through techniques such as laser desorption/ionization, achieving the construction of label-free, unbiased spatial proteomic maps (152). Additionally, digital spatial proteomics (such as the NanoString GeoMx platform) combines immunofluorescence pre-staining and region selection to enable high-throughput quantitative analysis of multiple proteins within areas of interest, providing powerful tools for the study of tumor heterogeneity and immune microenvironments (153).

These spatial proteomics technologies have outstanding capabilities for analyzing the spatial distribution of protein expression. For example, MSI can accurately distinguish different microenvironments such as tumor areas, stromal areas, and infiltrating immune cell areas within tumor tissues, revealing specific protein expression characteristics in each region. For instance, MSI differentiated between tumor and stromal regions in bladder cancer tissues, finding that the tumor area was predominantly composed of nuclear proteins such as histone H2A, while the stromal area was rich in collagen peptides. The presence or absence of muscle layer infiltration could be distinguished by markers like keratin 7 and vimentin, and these results were validated through immunohistochemistry (152). Digital spatial proteomics further enhances spatial resolution and quantitative capabilities, allowing for the selection of tumor-enriched areas, immune cell-enriched areas, tumor-immune interfaces, and multiple regions of interest (ROIs) in solid tumors such as bladder cancer based on immunofluorescence markers, quantitatively analyzing the expression of various proteins including PD-L1, Ki-67, HLA-DR, and HER2, revealing the spatial heterogeneity of protein expression and its association with treatment responses (153, 154). Such spatial distribution information is crucial for understanding phenomena like immune cell infiltration, immune exhaustion, and immune rejection in immune microenvironment studies.

Spatial proteomics technology has also propelled the discovery of tumor heterogeneity and spatial biomarkers (155). By providing high-dimensional spatial protein expression maps, it can identify spatial protein characteristics associated with tumor progression, treatment responses, and prognosis. For example, spatial proteomics has revealed the distribution and functional states of immune cells in different regions of bladder cancer tissues, discovering that the expression of markers like PD-L1 and Ki-67 in different spatial regions is closely related to the efficacy of neoadjuvant chemotherapy and immune checkpoint inhibitors (153, 156). This spatial dependency suggests that relying solely on overall protein expression levels may underestimate key biological features, while spatial proteomics can more accurately guide the formulation of personalized treatment strategies. The high resolution and multidimensional capabilities of spatial proteomics make it an ideal tool for elucidating the complexities of the tumor immune microenvironment, thus promising to drive the development of new immunotherapy targets and predictive biomarkers.

In summary, spatial proteomics technology, through high-throughput, multiplexing, and spatial resolution capabilities, provides unprecedented tools for the in-depth study of the immune microenvironment in bladder cancer. With the continuous advancement of technology and expansion of applications, spatial proteomics is expected to play a greater role in revealing the biological mechanisms of tumors, guiding precise immunotherapy, and discovering novel biomarkers.

#### 4.1.3 Multi-omics joint spatial analysis

Multi-omics joint spatial analysis is an important frontier in the current research of the immune microenvironment in bladder cancer. By integrating multi-dimensional data such as spatial transcriptomics and proteomics, it can comprehensively reveal the complex relationships between cell types, molecular expressions, and spatial distributions within tumor tissues. For example, researchers have utilized single-cell RNA sequencing, spatial transcriptomics, proteomics, and immunohistochemistry to explore the spatial distribution and functional status of tertiary lymphoid structures (TLS) and their associated immune cells in bladder cancer. They found that high levels of B cells and TLS are closely related to a good prognosis in patients with MIBC. This finding relies not only on the spatial transcriptomics for locating immune cells in TLS regions and analyzing their gene expression profiles but also confirms the secretion of key cytokines and intercellular interactions through immunohistochemistry (157). The combined application of spatial omics and proteomics allows for the quantification and visualization of spatial heterogeneity of immune cell subpopulations, cytokines, and functional molecules such as antibodies, thereby helping to reveal the dynamic changes in immune responses within the tumor microenvironment.

Moreover, multi-omics spatial analysis is not limited to the localization of immune cells; it can also uncover the spatial interaction mechanisms between tumor cells and immune cells. Novel immune-suppressive molecules represented by S100A5 and LRFN2 were found through spatial transcriptomics combined with proteomics analysis, where their high expression in tumor cells was closely related to the spatial exclusion phenomenon of CD8+ T cells. This type of spatial exclusion relationship has been validated at both the protein and transcriptional levels, further revealing the mechanism by which tumor cells limit the infiltration and functional activation of effector T cells by suppressing the secretion of chemokines (158, 159). This multi-omics spatial joint strategy not only reveals key molecules involved in tumor immune evasion but also provides a theoretical basis for combination immunotherapy targeting these molecules. The synergistic analysis of spatial transcriptomics and proteomics can more accurately depict the spatial dynamics of the tumor immune microenvironment, especially in elucidating the distribution relationship between immune-suppressive molecules and immune cells.

Furthermore, the integration of spatial omics with emerging technologies such as deep learning endows multi-omics spatial analysis with higher resolution and automation capabilities. By performing spatial segmentation of pathological slices and automatically identifying immune cell aggregation areas, researchers can quantify the distribution ratios of immune cells within and around tumor tissues, and conduct joint analyses with multi-omics data (e.g., transcriptomics, proteomics). They discovered that the proportion of lymphocyte aggregation within the tumor (Lymph_inside %) is significantly positively correlated with the abundance of anti-tumor immune cells and the activity of apoptotic pathways (160). This multi-omics spatial joint analysis strategy is expected to promote the development of novel prognostic biomarkers based on spatial distribution characteristics.

In summary, multi-omics joint spatial analysis provides an unprecedented multi-dimensional interpretation of the complexity of the immune microenvironment in bladder cancer, greatly advancing the research progress on tumor immune heterogeneity and spatial functional mechanisms. This strategy not only lays a solid foundation for precise prognostic assessments and personalized immunotherapy but also points the way for subsequent explorations of the dynamic evolution of the tumor microenvironment and its regulatory mechanisms.

### 4.2 Spatial heterogeneity characteristics of the bladder cancer immune microenvironment

#### 4.2.1 Spatial distribution patterns of immune cells

The application of spatial omics techniques has greatly enriched our understanding of the spatial distribution characteristics of various immune cells in the immune microenvironment of bladder cancer. Through methods such as spatial transcriptomics, spatial proteomics, and multiplex immunofluorescence, researchers can analyze the localization characteristics of major immune cells such as T cells, macrophages, and dendritic cells at the tissue section level. For instance, in muscle-invasive bladder cancer and NMIBC, CD8+ T cells often exhibit a pattern of aggregation at the tumor edge or within the tumor, and their density and distribution are closely related to patient prognosis. Spatial proteomics data indicate that the distribution of T cells within tumors can be categorized as “excluded,” “infiltrated,” or “desert,” which helps in understanding tumor heterogeneity and personalized treatment (161).

The spatial distribution of macrophages in the bladder cancer microenvironment also shows high heterogeneity. The immunosuppressive subtype represented by CD163+ M2 macrophages is found to be more abundant in high-grade tumors in female patients, closely associated with immune tolerance and increased recurrence risk (162). Spatial transcriptomics combined with single-cell sequencing further reveals a complex spatial interaction network between macrophages, tumor cells, and T cells, where specific subgroups of macrophages (such as SPP1+ type) promote immune evasion and resistance formation through bidirectional signaling with cancer stem cells (163). This phenomenon of spatial co-localization suggests that macrophages not only participate in immune suppression but may also directly influence the stemness and treatment response of tumor cells.

Dendritic cells (DCs) in bladder cancer are primarily distributed at the interface between the tumor and stroma, especially at the epithelial-stromal junction, where a high interface density ratio (IDR) of CD11c+ dendritic cells is associated with longer recurrence-free survival (164). Such spatial distribution characteristics may reflect the key role of dendritic cells in antigen presentation and T cell activation, and their proximity to T cells and macrophages influences the activation state of the local immune microenvironment. Notably, spatial omics analysis also indicates that the spatial distribution of dendritic cells is closely related to clinical indicators such as tumor stage and treatment response, suggesting their potential value as prognostic and immunotherapy response biomarkers.

The spatial interaction relationships between different immune cell subgroups are another focus of spatial omics research. Techniques such as spatial transcriptomics and imaging mass spectrometry reveal that T cells, B cells, dendritic cells, and macrophages form multicellular “ecological niches” in the tumor microenvironment, such as tumor-associated tertiary lymphoid structures, where the enriched T, B cells, and plasma cells are closely linked to tumor antigen-specific immune responses (157, 165). The spatial distribution of TLS is often observed at the tumor edge or stroma, and its maturity and spatial organizational structure are closely related to the strength of anti-tumor immunity and patient prognosis. Spatial omics also reveals complex interaction regulation between tumor cells, cancer-associated fibroblasts, and immune cells through spatial proximity (150, 166).

The continuous advancement of spatial omics technologies allows us to depict the spatial distribution and interaction networks of immune cells at single-cell and even subcellular levels. With improved spatial resolution and multi-omics integration capabilities, future studies are expected to reveal more dynamic distribution patterns of immune cells in microenvironments and their impact on the occurrence, progression, and treatment response of bladder cancer, providing a theoretical basis for the formulation of precise immunotherapy strategies.

#### 4.2.2 Spatial interactions between tumor cells and immune cells

Spatial omics studies in the tumor immune microenvironment have revealed complex spatial interaction patterns between tumor cells and immune cells. By employing multi-omics techniques such as spatial transcriptomics and spatial proteomics, researchers are able to analyze the localization, proximity relationships, and functional states of tumor cells and various immune cells within tumor tissues at single-cell or even subcellular levels. For instance, spatial transcriptomics analysis shows that in recurrent tumors of bladder cancer, although the overall number of immune cells may not increase, the spatial interactions between tumor epithelial cells and immune cells are significantly enhanced, suggesting that tumor cells influence the immune microenvironment by regulating spatial contact with immune cells (150). Additionally, spatial omics has revealed the heterogeneity of immune cell infiltration and distribution among different tumor subtypes, which is closely related to treatment response and prognosis (75).

Spatial expression of immune evasion-related molecules in tumor cells shows specific distribution patterns in bladder cancer. Spatial omics techniques have confirmed that surface immune checkpoint molecules (such as PD-L1) and stem cell characteristic-related molecules (such as IGF2BP3, SPHK1) in tumor cells are often spatially adjacent to exhausted CD8+ T cells, forming “immunosuppressive hotspots.” The presence of these regions is associated with an increased proportion of patients who respond well to immunotherapy (167). Further spatial proteomics and multiplex immunofluorescence analyses have found that resistant tumor cells form a multicellular microenvironment spatially with macrophages, promoting resistance through PARP14-related mechanisms (151). The application of spatial omics allows us to more accurately locate the expression regions of immune evasion-related molecules and their spatial relationships with immune cells, providing a theoretical basis for the discovery of personalized immunotherapeutic targets.

The spatial aggregation of immunosuppressive cell populations (such as TAMs, regulatory T cells, M2 macrophages, etc.) in the bladder cancer microenvironment has also been revealed by spatial omics techniques. Studies have shown that TAMs are highly concentrated in the core regions of tumors and interact spatially with T cells through immunosuppressive signaling axes such as SPP1-CD44 and NECTIN2-TIGIT, inhibiting the function of CD8+ T cells and forming immunosuppressive microregions (168). Furthermore, spatial omics has also discovered that CD163+ M2 macrophages and PD-L1+ immune cells are more abundant in tumors of female bladder cancer patients, and their high-density aggregation is closely linked to poor prognosis (162). In cases of bladder cancer resistance or recurrence, immunosuppressive cells spatially co-localize with tumor stem-like cells, synergistically promoting tumor immune evasion and treatment resistance (163). Functional studies of these spatial aggregation areas reveal that immunosuppressive cell populations not only regulate T cell exhaustion and immune tolerance through the secretion of immunosuppressive factors but also weaken anti-tumor immune responses through direct spatial contact.

Spatial transcriptomics technology has revealed the impact of dynamic spatial interactions between tumor cells and immune cells on tumor progression and responses to immunotherapy. For example, the spatial communication between tumor-associated fibroblasts and immune cells in tumor tissue is significantly enhanced in recurrent bladder cancer (150). Additionally, the expression of certain metabolism-related molecules (such as FAAH) by tumor cells influences tumor activity in cancer-associated fibroblasts, and spatial transcriptomic analysis has uncovered the spatiotemporal heterogeneity of metabolic gene expression as well as the enrichment of immunosuppressive cells and immune checkpoint molecules (169). These findings emphasize that spatial transcriptomics can not only reveal static cell distributions but also dynamically capture the complex interaction networks between tumor cells and immune cells.

Overall, spatial transcriptomics technology has advanced our systematic understanding of the spatial structure and function of the immune microenvironment in bladder cancer. Tumor cells shape the immunosuppressive microenvironment by spatially regulating the expression of immune evasion molecules, inducing the aggregation of immunosuppressive cell populations, and forming complex spatial interaction networks with immune cells. These spatial interaction features provide new insights for discovering immunotherapy targets and predicting efficacy in bladder cancer, and suggest that future efforts should further integrate multimodal spatial transcriptomics data to deeply analyze their dynamic changes in different subtypes and therapeutic contexts.

#### 4.2.3 Spatial analysis of immune escape mechanisms: heterogeneous expression of immune checkpoint molecules and spatial accumulation of immunosuppressive cells

##### 4.2.3.1 Spatial expression characteristics of immune checkpoint molecules

In recent years, spatial omics technologies have provided powerful means to elucidate the localization and expression differences of immune checkpoint molecules in the immune microenvironment of bladder cancer (BLCA). The spatial distribution and expression patterns of key immune checkpoint molecules such as PD-1, PD-L1, and CTLA-4 exhibit significant heterogeneity in bladder cancer. For example, combined analysis of single-cell transcriptomics and spatial transcriptomics revealed that PD-L1 (CD274) is highly expressed in tumor stem cell-like cells (ALDH+ CSC-like cells) and their surrounding regions, which are closely associated with an immunosuppressive state and poorer survival prognosis in patients. Furthermore, spatial transcriptomics and CODEX multiplex immunofluorescence imaging further confirmed that IGF2BP3+SPHK1+ tumor cells are spatially closer to exhausted CD8+ T cells, suggesting that the expression of immune checkpoint molecules is influenced not only by cell type but also closely related to their microenvironmental localization (167).

Furthermore, there is an important correlation between the spatial expression of immune checkpoint molecules and the response to immunotherapy. Studies have found that MS4A4A+ TAMs are primarily distributed in the stromal areas of bladder cancer tissues, and their high expression is closely related to the upregulation of immune checkpoint molecules such as PD-1, LAG-3, and HAVcr-2. The TAMs in these regions co-localize with CD8+ T cells and Foxp3+ regulatory T cells, forming an immunosuppressive microenvironment that affects the efficacy of immunotherapy. The abundance of MS4A4A+ TAMs not only predicts poor prognosis but is also associated with decreased response to Bacillus Calmette-Guérin (BCG) immunotherapy, suggesting that the spatial distribution of immune checkpoint molecules may serve as one of the predictive indicators for the effectiveness of immunotherapy (99).

Gender differences also influence the spatial expression characteristics of immune checkpoint molecules. Research on non-muscle invasive bladder cancer shows that the expression of immune checkpoint molecules such as PD-L1, CTLA-4, and PD-1 is significantly higher in high-grade tumors in females than in males, and the spatially dense distribution of PD-L1+ cells with CD163+ M2 macrophages is associated with an increased risk of recurrence. This finding emphasizes the need to fully consider gender-related spatial expression differences in the clinical design of immunotherapy to optimize efficacy (162).

Spatial omics also reveal the co-expression of immune checkpoint molecules with other immune regulatory factors. For example, CD73 is highly expressed in CD8+ cytotoxic T cells and Foxp3+ regulatory T cells within bladder cancer tissues and shows a clear trend of co-expression with PD-1. The high infiltration of CD73+ Treg cells is independently associated with tumor progression and poor prognosis. Notably, CD73+ T cells and PD-L1+ cells often exhibit compartmentalized spatial distribution, suggesting that the two may regulate tumor immune evasion through different spatial microenvironment mechanisms (170).

In summary, spatial omics technology reveals the high spatial heterogeneity of immune checkpoint molecules in the immune microenvironment of bladder cancer and their complex interactions with immune cells. The spatial localization of immune checkpoint molecules not only affects the tumor immune suppression state but is also closely related to the response to immunotherapy, providing important evidence for the formulation of precise immunotherapy strategies.

##### 4.2.3.2 Spatial aggregation and function of immunosuppressive cells

The spatial distribution and function of immunosuppressive cells in the tumor microenvironment of bladder cancer have received increasing attention. Spatial transcriptomics techniques reveal that the spatial aggregation of immunosuppressive cells, such as regulatory T cells and myeloid-derived suppressor cells (MDSC), in tumor tissues exhibits high heterogeneity and is closely related to tumor progression and responses to immunotherapy. For example, combined analysis of single-cell RNA sequencing and spatial transcriptomics shows that immunosuppressive cells are enriched in tumor regions of bladder cancer patients with high TMs (taurine metabolism abnormality index), accompanied by high expression of immune checkpoint molecules, suggesting their central role in immune evasion (169). Furthermore, spatial transcriptomics further reveals that these cells display different distribution patterns in the tumor margin and central region, with some areas forming “clusters” of immunosuppressive cells, which are often closely associated with the activation of signaling pathways related to active proliferation and metastasis of tumor cells.

In terms of the mechanisms underlying the formation of the immunosuppressive microenvironment, research has found that multiple signaling pathways and intercellular interactions jointly drive the enrichment and functional enhancement of Tregs and MDSCs. High-throughput spatial omics and single-cell omics analyses reveal that bladder cancer-associated fibroblasts influence tumor cell activity by secreting chemokines (such as CCL15) and are associated with immunosuppression (169, 171). The immunosuppressive function of MDSCs is also regulated by cytokines secreted by tumor cells (such as IL-6) and the activation of MAPK signaling pathways they mediate, enhancing their ability to inhibit T cell proliferation and function (172). Spatial omics evidence shows that the expression of these signaling molecules exhibits high spatial heterogeneity in the tumor microenvironment, determining the local dense distribution and functional status of immunosuppressive cells.

In addition to Tregs and MDSCs, TAMs and regulatory B cells (Bregs) in the bladder cancer TIME are also closely related to the spatially immunosuppressive microenvironment. Spatial transcriptomics and multiplex immunofluorescence analyses show that CD163+ macrophages are enriched in high-grade tumors in female patients and are spatially co-localized with PD-L1+ cells, with their high density significantly associated with an increased risk of recurrence (162). In high-grade bladder cancer tissues, immunosuppressive B cells (such as IL-10+, TGF-β+ Bregs) form local enrichments within tumor tissues, promoting the maintenance of the TIME immunosuppressive state (173).

Spatial omics also reveals a complex interaction network between immunosuppressive cells and other TIME components such as tumor cells and fibroblasts. For example, CAFs promote infiltration in bladder cancer by regulating TGFβ1, forming an immunosuppressive microenvironment (171). Additionally, tumor cell metabolic reprogramming (such as taurine metabolism, glucose metabolism, etc.) can inhibit immune cell function through metabolic competition, further promoting tumor immune evasion (169, 174). On a spatial level, these mechanisms lead to the formation of functional “immune cold zones” by immunosuppressive cells within tumor tissues, limiting the infiltration and anti-tumor activity of effector T cells.

In summary, spatial omics technology provides a powerful tool for analyzing the spatial aggregation of immunosuppressive cells in the bladder cancer TIME and their functional mechanisms. Through multi-omics integration, the spatial heterogeneity of immunosuppressive cells such as Tregs, MDSCs, TAMs, and Bregs in the tumor locality and their close association with tumor progression and immune therapy resistance have been revealed. In the future, research based on spatial omics is expected to promote the development of precise intervention strategies targeting the immunosuppressive microenvironment, thereby enhancing the clinical benefits of immune therapy for bladder cancer.

## 5 Integration of radiomics and spatial omics: from technological convergence to clinical translation

**Figure 2:**
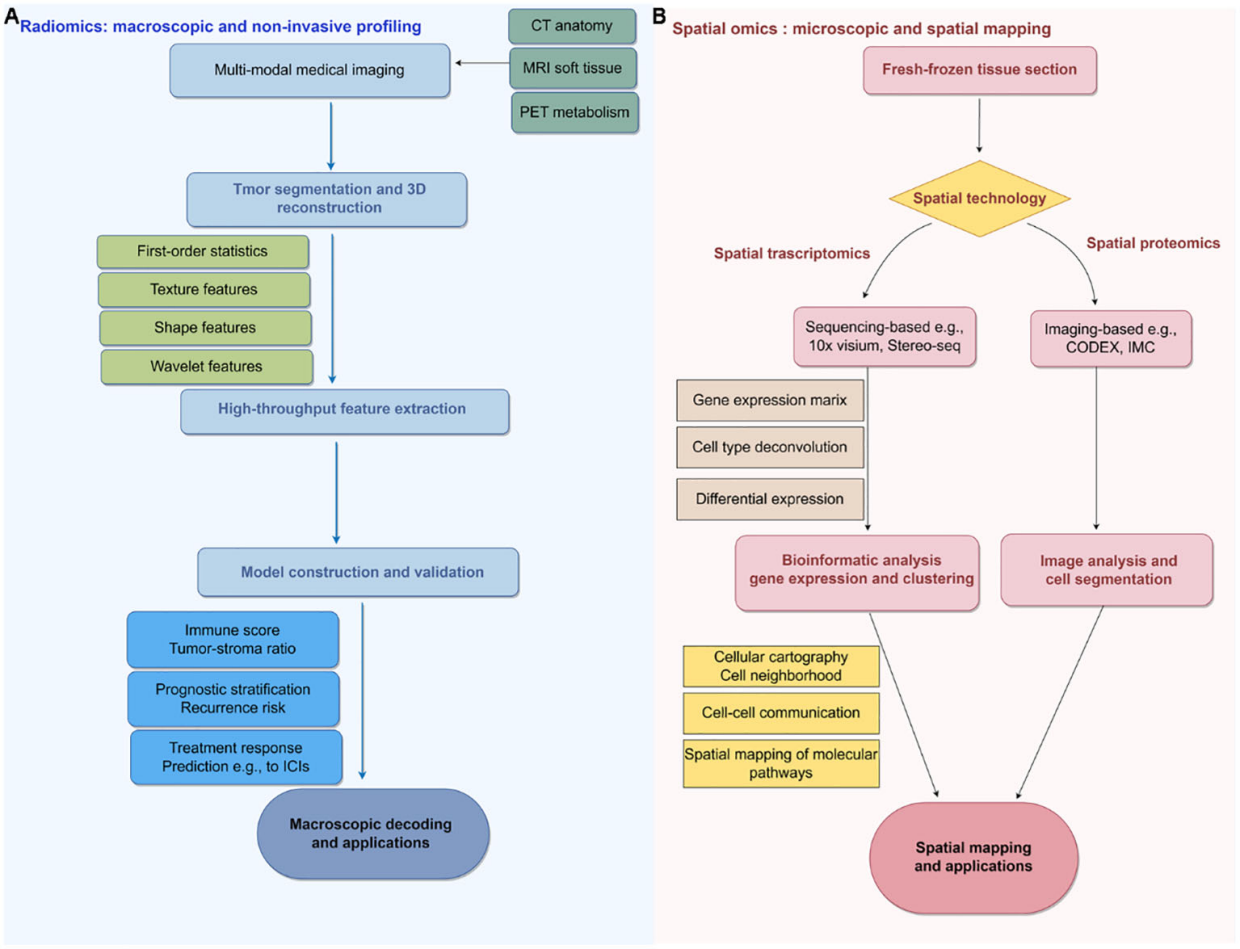
Workflows of radiomics and spatial omics in deciphering the bladder cancer TIME. **(A)** The radiomics workflow begins with multi-modal medical imaging (CT, MRI, PET). Following tumor segmentation and 3D reconstruction, high-throughput quantitative features (e.g., shape, texture) are extracted. These features are used to build machine learning models for non-invasive applications such as predicting immune cell infiltration, patient prognosis, and response to therapy (e.g., Immune checkpoint inhibitors, ICIs). **(B)** The spatial omics workflow begins with a fresh-frozen tissue section, which is processed using either sequencing-based (e.g., Spatial transcriptomics) or imaging-based (e.g., Spatial proteomics) platforms. Subsequent bioinformatic or image analysis generates high-resolution maps of the TIME, enabling applications such as cellular cartography, analysis of cell-cell interactions, and spatial mapping of active molecular pathways.

The high heterogeneity of the immune microenvironment in bladder cancer poses a major challenge for achieving precise immunotherapy. As two cutting-edge technologies, radiomics and spatial omics provide important means to elucidate tumor complexity from macro imaging characterization and micro molecular spatial dimensions, respectively. However, both techniques have significant limitations when applied separately: radiomics can non-invasively capture the overall heterogeneity of tumors, but the imaging features it extracts often lack clear biological interpretation, forming a “black box” model; spatial omics, while finely depicting the spatial distribution of cells and molecules, relies on invasive biopsies, which can introduce sampling bias, and it is difficult to achieve dynamic monitoring during treatment. Therefore, the integration of radiomics and spatial omics to construct a bridge from macro imaging features to micro spatial biological mechanisms has become an inevitable choice to promote bladder cancer precision medicine into a new phase, and it is also an important pathway for the clinical translation of “virtual biopsy.” Here, **Figure 2** outlines the complementary workflows of these two technologies, which together form the basis of this integrated approach, and we also summarized the key studies of utilization of radiomics and spatial omics in bladder cancer (21, 135, 140, 150, 152, 153, 157–159, 162, 164, 167, 168, 171, 175, 177) (**Table 1**).

**Table 1:** Summary of radiomics–spatial omics studies in bladder cancer TME: from modality to translation.

Modality/ platform scope	TME component/biological target	Clinical & translational application	Representative findings & references
Radiomics			
Multi-omic Spatial Profiling (e.g., spatial transcriptomics, proteomics, metabolomics)	Tumor-stroma interface, tertiary lymphoid structures (TLS), perineural invasion	Characterization of immune evasion mechanisms; biomarker identification for combination therapy	Spatial technologies reveal immune exclusion zones, TLS composition, and nerve–tumor interactions driving resistance. Review emphasizes integration of spatial multi-omics for clinical translation (21).
Contrast-enhanced CT + Radiomics (full-volume 3D segmentation, IBSI-standard features)	Tumor stage (organ-confined T2 vs. extravesical T3); Post-TURB tissue changes	Preoperative staging for treatment planning; optimizing imaging timing for staging accuracy	The radiomics model improved performance when CT was performed >14 days post-TURB (AUC 0.80) vs. ≤14 days (AUC 0.68). Adding clinical biomarkers (creatinine, hemoglobin, etc.) further enhanced AUC to 0.82 (delayed imaging). This suggests delaying imaging after TURB reduces post-surgical artifact and improves radiomic feature stability for staging (158).
Multimodal imaging (CT, MRI, PET) + subregion segmentation (habitat/clustering)	Intra-tumoral heterogeneity (imaging habitats, subregional imaging phenotypes)	Improving radiomics model performance for cancer diagnosis, treatment response prediction, and prognosis	This review systematically summarizes the limitations of single-modality whole-tumor radiomics (information loss, heterogeneity dilution) and demonstrates that multimodal imaging combinations and subregion-based feature extraction can capture complementary information and preserve intra-tumoral heterogeneity, leading to superior model performance across various cancers (153).
PET/CT + PET/MRI (with [^18^F]FDG, immunoPET tracers)	Lymph node and distant metastases; PD-L1 expression (via immunoPET)	Precise staging (N/M staging); treatment selection for immunotherapy	PET/CT outperforms conventional CT for nodal metastasis detection. PET/MRI has higher soft-tissue contrast but limited sensitivity for early-stage BCa due to renal FDG excretion. ImmunoPET targeting PD-L1 shows high uptake in PD-L1+ tumors, potentially guiding immunotherapy selection (150).
Multimodal imaging (CT, MRI, PET) + subregion segmentation (habitat/clustering)	Intra-tumoral heterogeneity (imaging habitats, subregional imaging phenotypes)	Improving radiomics model performance for cancer diagnosis, treatment response prediction, and prognosis	This review systematically summarizes the limitations of single-modality whole-tumor radiomics (information loss, heterogeneity dilution) and demonstrates that multimodal imaging combinations and subregion-based feature extraction can capture complementary information and preserve intra-tumoral heterogeneity, leading to superior model performance across various cancers (152).
Multimodality imaging (CT, MRI, CBCT, PET/CT) + Radiomics preprocessing pipeline (resampling, normalization, discretization)	Feature robustness & reproducibility (intrinsic property of the radiomic workflow)	Standardizing radiomic analysis methodology; Enhancing reliability of radiomic biomarkers for clinical translation	Systematic review of 43 studies: voxel resampling (44% of studies), normalization, and discretization critically influence feature stability. ICC is the most used metric (58% of MRI studies). From 2021, studies began selecting preprocessing parameters based on model performance. Standardized preprocessing is essential for reproducible feature extraction (162).
High-definition CT + Radiomics (Cox/Lasso feature selection) + Bulk RNA-Seq + Deep Residual Neural Network	Tumor stage (pathological stage); Prognostic radiomics signature (3 features) & gene signature (4 genes)	Preoperative staging prediction; Long-term prognosis (1/3/5-year OS) via integrated nomogram	Combined radiomics-gene nomogram achieved AUCs of 0.870 (1-year), 0.873 (3-year), and 0.971 (5-year). A 3-factor radiomics signature and a 4-gene signature were identified as independent predictors, highlighting the synergy of imaging and transcriptomic features for staging and prognosis (159).
Multicentric MRI (T1w-gd, T2w-flair) + Radiomics harmonization (ComBat & AutoComBat)	Cross-center data heterogeneity/Batch effects (intrinsic property of the radiomic workflow)	Enabling generalizable radiomic signatures for brain tumor grading; supporting multi-institutional clinical deployment	AutoComBat automatically determines batch labels via constrained clustering using MRI metadata or quality metrics. On a 8-center glioma dataset: Preprocessing performed best for T1w-gd grading task;AutoComBat;AutoComBat (metadata ± quality metrics) outperformed conventional ComBat for T2w-flair grading, demonstrating its potential for automatic data harmonization (167).
Multiparameter MRI (T1WI, T2WI, DWI, DCE) + Radiomics (intratumoral & peritumoral regions)	Ki67 proliferation index; Histological grade (tumor differentiation)	Preoperative non-invasive prediction of tumor proliferation and malignancy grade; aiding clinical decision-making	The MP-MRI radiomics model achieved high AUCs for Ki67 (0.977 training, 0.852 test) and histological grade (0.972 training, 0.710 test). Feature selection (mRMR for Ki67, LASSO for grade) and segmentation region (intratumoral vs. peritumoral) differentially impacted model performance (157).
T2w-MRI (pelvic region, prostate cancer) + Radiomics preprocessing (intensity normalization methods)	Feature reproducibility & reliability (intrinsic property of the radiomic workflow)	Standardizing MRI radiomic preprocessing; improving reproducibility of imaging biomarkers for clinical translation	Compared three normalization methods: Norm_Mean, Norm_ROI (by bladder urine), Norm_HM (histogram matching). Norm_HM outperformed Norm_Mean for first-order features (ICC 0.76 vs. 0.33). Only a small subset of features (skewness, kurtosis, correlation, select GLCM features) remained reproducible across all conditions (164).
Spatial Omics			
Spatial omics technologies: Spatial Transcriptomics (ST) + Spatial Proteomics (SP)	Tumor microenvironment components: immune cells, fibroblasts, blood vessels, ECM, signaling molecules	Characterizing TME heterogeneity; uncovering spatial organization of immune cells; guiding precision immunotherapy	This review systematically summarizes the principles, applications, and progress of ST and SP technologies in TME research. ST enables visualization of gene expression within tissue context, revealing immune cell infiltration patterns and tumor-immune interactions. SP (e.g., multiplex IHC, imaging mass cytometry) provides spatial mapping of protein expression at single-cell resolution, enabling in-depth characterization of the TME (168).
Transcriptomics (RNA-seq) + Multiplex immunofluorescence	CD163+ M2 macrophages, CD79a+ B cells, PD-L1 immune checkpoint	Prognostic stratification by sex; informing immunotherapy trial design	In NMIBC, tumors from female patients had higher infiltration of PD-L1+ cells and CD163+ M2-like macrophages. High abundance of CD163+ macrophages and CD79a+ B cells correlated with decreased recurrence-free survival. This suggests sex-based differences in the TME that may affect immunotherapy response (135).
scRNA-seq + Spatial RNA-seq (10x Visium) + Multiplex IHC	MYBL2hi Cancer Stem Cells (CSCs), SPP1+ Macrophages, CCL3+ Neutrophils; Key signaling: CCL15-CCR1, SPP1-ITGα9β1	Predicting & reversing resistance to neoadjuvant immune checkpoint blockade (nICB)	In non-responders to nICB, MYBL2hi CSCs were enriched and communicated with SPP1+ macrophages. CSC-derived CCL15 induced SPP1 upregulation in macrophages, which reciprocally enhanced cancer stemness and resistance via the SPP1-ITGα9β1 axis. Combined MYBL2 knockdown and SPP1 targeting synergistically enhanced ICB efficacy *in vivo* (140).
Multiplex IHC (Digital Pathology)	CD11c+ (dendritic/myeloid cells), CD8+ T cells, ICOS+ T cells; spatial feature; Epithelial-Stromal Interface Density Ratio (IDR)	Predicting recurrence of NMIBC after BCG immunotherapy; risk stratification	Higher IDR of CD11c, CD8, and ICOS was associated with longer recurrence-free survival. The CD11c IDR was the most informative predictor, and its combination with re-TUR history or tumor stage yielded models with high concordance (C-index >0.7) (139).
scRNA-seq + Spatial Transcriptomics (10x Visium)	Tertiary Lymphoid Structures (TLSs), IGHA1+ & IGHG1+ plasma cells; Tumor microenvironment composition: iCAF, macrophages, NK cells	Predicting response to anti-PD-L1 immunotherapy and cisplatin-based adjuvant chemotherapy in MIBC	Identified an IGHA1^low/IGHG1^high subgroup associated with an antitumor immune microenvironment (high immune effector cells) and better response to both PD-L1 inhibitors and chemotherapy. Spatial analysis revealed IGHG1 clonotypes mature within TLSs and disseminate into the tumor bed after treatment, potentially cooperating with iCAF, macrophages, and NK cells (175).
scRNA-seq + Spatial Transcriptomics	MHC-II^+^ cancer cells (induced by IFN-γ), CD8^+^ T cells, Tregs, SPP1^+^ macrophages; Key signaling: IFN-γ	Understanding immune evasion mechanisms in MIBC; potential target for immunotherapy	MHC-II^+^ cancer cells were enriched in MI tumors, induced by IFN-γ, and exhibited enhanced proliferation/migration. They spatially colocalized with CD8^+^ T cells, Tregs, and SPP1^+^ macrophages, engaging inhibitory receptors to promote CD8^+^ T cell exhaustion and immune evasion (161).
scRNA-seq + Spatial Transcriptomics (ST) with deconvolution	Cancer-associated fibroblasts (CAFs), malignant epithelial cells, NK/T cells; Key interaction: CAF-malignant cell & CAF-immune cell crosstalk	Understanding mechanisms of bladder cancer recurrence; identifying CAF-related therapeutic targets	Compared primary vs. recurrent bladder tumors (6 samples). Recurrent tumors have higher intratumoral heterogeneity. Spatial communication analysis revealed markedly increased CAF-malignant cell and CAF-immune cell interactions in recurrent tumors, a finding first observed at the spatial level (171).
Multi-omics: bulk RNA-seq, scRNA-seq, ProcartaPlex immunoassays, functional experiments, TissueFAXS spatial analysis	LRFN2 (leucine-rich repeat and fibronectin type-III domain-containing protein 2); CD8+ T cell infiltration, proliferation, and differentiation; pro-inflammatory chemokines	Identifying LRFN2 as a novel immunosuppressive target to overcome ICI resistance; predicting immunotherapy response	LRFN2 is specific to BLCA and shapes aLRFN2 non-inflammatory TME. It inhibits CD8+ T cell recruitment and functional transition by reducing pro-inflammatory chemokine secretion. Spatial analysis revealed exclusivity between LRFN2+ tumor cells and CD8+ T cells. LRFN2 knockdown enhanced ICI efficacy in preclinical models. Clinically, LRFN2 predicts immunotherapy responses (176).
Mass Cytometry (CyTOF) + Imaging Mass Cytometry (IMC); single-cell proteomics with spatial architecture	Cancer stem-like cell cluster (ALDH+PD-L1+ER-β-); immune cell spatial architectures (excluded, infiltrated, deserted); tumor-associated collagens (curved, stretched, directional, chaotic)	Identifying novel prognostic cancer stem cell markers; stratifying MIBC patients based on TME spatial patterns; guiding personalized therapy	First study providing in-depth single-cell level insight into MIBC TME complexity. Identified a previously overlooked ALDH+PD-L1+ER-β- cancer stem-like cell cluster strongly associated with poor prognosis. Elucidated three immune cell spatial architectures (excluded, infiltrated, deserted) and 和four tumor-associated collagen architectures (curved, stretched, directional, chaotic) in the MIBC TME (177).

### 5.1 Core challenges of data fusion and AI-driven methodologies

#### 5.1.1 Core challenges and strategies for data fusion

The primary challenge in fusing macroscopic radiomics with microscopic spatial omics lies in the inherent gap in scale and dimensionality between the two. Radiomic features are derived from millimeter-scale voxels, each containing millions of cells, reflecting the overall average information of a tissue region (178, 179). In contrast, spatial omics data operate at micron-to-single-cell resolution, capable of revealing the spatial distribution of cell communities and molecular expression profiles (180–182). These substantial differences in spatial scale, data dimensionality, and physical significance make direct fusion difficult. To address this, fusion strategies are primarily categorized into early, intermediate, and late fusion. Intermediate fusion is currently the more feasible strategy; it allows feature extraction from both data modalities separately before correlating and integrating them at the feature level, thereby circumventing the issue of mismatched raw data scales (183–185). The technical prerequisite for achieving fusion is spatial registration and alignment, which requires precisely registering ex vivo spatial omics slices (2D) to the corresponding anatomical locations of in vivo medical images (3D). This process typically involves a multi-step workflow: for instance, first co-localizing H&E-stained digital pathology images with spatial omics data, and then utilizing deep learning-based pathology-image registration networks to map the digital pathology images onto the corresponding slice planes of MRI or CT, thereby establishing a spatial coordinate correspondence from the microscopic to the macroscopic scale (186, 187). Such precise spatial association forms the foundation for subsequent meaningful cross-scale feature mapping and biological interpretation.

#### 5.1.2 Specific AI architectures and feature mapping processes

Achieving cross-scale data fusion relies on meticulously designed AI architectures. The following are three mainstream technical pathways currently in use:

1. Cross-modal alignment based on graph neural networks (GNN)

GNNs are inherently capable of handling non-Euclidean spatial data, making them particularly suitable for processing cellular neighborhood relationships (188, 189). In the fusion of imaging and spatial omics, radiomic features (such as texture and morphological features extracted from MRI) and spatial transcriptomics data (such as gene expression profiles and cellular neighborhood relationships) can be constructed as graph structures separately. By designing a Cross-modal Graph Contrastive Learning framework, the model can learn mapping functions that align these two graphs within a latent representation space (190, 191). Through message-passing mechanisms, GNNs aggregate neighbor information to learn embedding representations for each cell or tissue region, thereby capturing the complex spatial interactions within the tumor microenvironment (192, 193). For instance, a study on breast cancer integrated longitudinal MRI spatial habitat radiomics, transcriptomics, and single-cell RNA sequencing data to construct a multimodal model. This model captured the dynamic evolution of tumor spatial heterogeneity during neoadjuvant therapy, demonstrating high predictive efficacy (with AUC up to 0.888) in external validation and multi-omics cohorts, while revealing associations between imaging spatial features and enhanced immune activity (such as B-cell infiltration) (194, 195). This framework is equally applicable to bladder cancer, enabling the establishment of quantitative links between immune “hotspots” or “cold spots” defined by spatial omics and specific texture features in radiomics.

2. Transformer-based multimodal attention fusion

The self-attention mechanism within the Transformer architecture can effectively process multimodal sequential data (196). We can treat radiomics feature vectors and spatial omics gene expression matrices as two independent modal inputs, achieving feature interaction through cross-modal attention layers (197). Specifically, macroscopic imaging features can ‘query’ the most relevant parts of microscopic spatial omics features, and vice versa, thereby learning soft alignment and weight allocation between the two modalities (198). For instance, the model can learn that when a specific spatial region (such as the tumor invasive margin) highly expresses cytotoxic CD8+ T cell markers (e.g., GZMB, PRF1), the corresponding radiomics features for that region (e.g., high entropy, low uniformity) should be assigned higher weights. Visualizing these attention weights not only achieves fusion but also provides interpretability for model decisions, clarifying which imaging features correspond to which molecular spatial features (199–201).

3. Generative adversarial network (GAN)-based virtual staining and spatial super-resolution

GANs excel in image-to-image translation tasks (202, 203). In imaging-spatial omics fusion, GANs can be employed for “virtual staining”—generating “virtual immunohistochemistry” images with spatial biological significance by learning the mapping from conventional CT/MRI images to distribution maps of specific spatial molecular markers (e.g., PD-L1, CD8) (204, 205). For example, researchers have successfully predicted bladder cancer PD-L1 expression status based on CT radiomics features. Furthermore, incorporating prior knowledge from spatial transcriptomics, GANs can also enhance the spatial resolution of radiomics features, enabling non-invasive inference of cell neighborhood interaction patterns within the tumor microenvironment (206).

4. Joint embedding learning and dynamic monitoring

By designing dual-stream networks and utilizing contrastive or adversarial learning, data from the same patient but different modalities can be mapped into a shared latent semantic space. In this space, associated macro-micro feature pairs are positioned close to each other, thereby establishing intrinsic connections (207, 208). Additionally, for dynamic monitoring during treatment, spatial omics can be correlated with pre- and post-treatment changes in radiomics (i.e., delta-radiomics, such as tumor regression patterns and emerging heterogeneous regions) via temporal AI models (e.g., LSTM or Transformer) to capture the spatiotemporal evolution of the tumor microenvironment (209, 210).

#### 5.1.3 Explainable AI and clinical validation

To dismantle the “black box” nature of AI models and enhance their clinical credibility, Explainable AI (XAI) and feature map visualization are indispensable (211–213). This necessitates the adoption of techniques such as Grad-CAM and attention weight visualization to reveal which macroscopic imaging regions (e.g., specific enhancing sub-regions of a tumor), which features (e.g., specific texture patterns), correlate most strongly with which microscopic spatial characteristics (e.g., the formation of immune-excluded zones or specific gene expression clusters) (214, 215). Such visualization not only validates model reliability but also generates novel biological hypotheses; for instance, specific imaging texture patterns may directly map to cellular spatial communities within the tumor possessing immunosuppressive properties (216, 217). At the clinical level, attention heatmaps generated by XAI can directly guide decision-making: for example, regions with high attention weights corresponding to “immune-excluded zones” defined by spatial omics suggest that the patient may be insensitive to immune checkpoint inhibitors, thereby avoiding ineffective treatment (218).

Regarding clinical translation, challenges remain, including insufficient model generalization capabilities and decreased performance in external validation (219, 220). Future efforts require multi-center, prospective studies combined with standardized imaging acquisition and processing workflows to ensure model robustness across different clinical scenarios (221, 222). Furthermore, modeling that integrates radiomics with clinical variables can enhance predictive capability, offering new avenues for clinical model translation (223, 224).

### 5.2 Integrated application: realization and validation of “Virtual biopsy”

**Figure 3:**
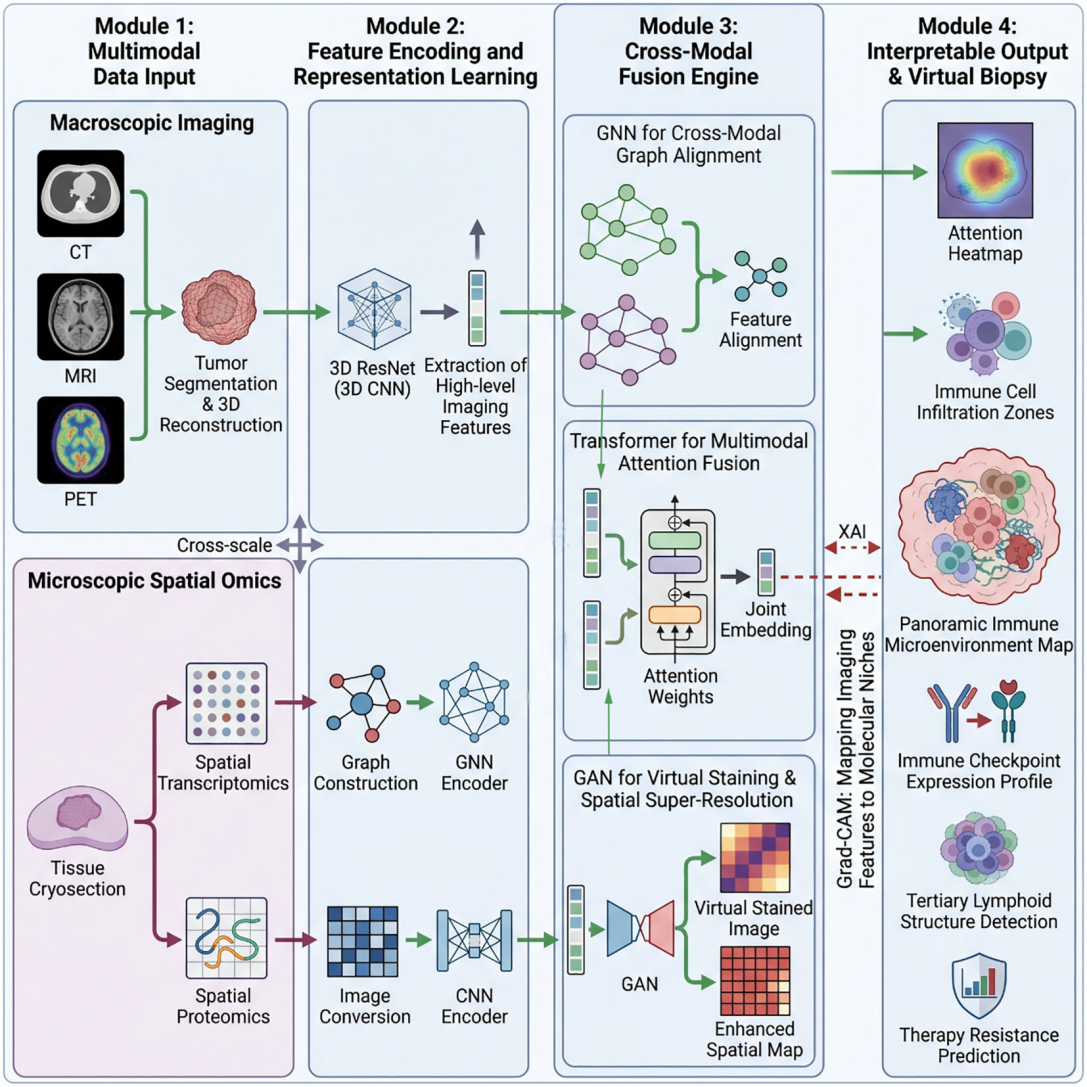
An integrated radiomics-spatial omics paradigm for “virtual biopsy” in bladder cancer. The workflow comprises four key modules (1): Multimodal data input, including macroscopic imaging (CT/MRI/PET) with tumor segmentation and 3D reconstruction, and microscopic spatial omics (spatial transcriptomics/proteomics) from tissue sections (2). Feature encoding and representation learning, where 3D CNNs (e.g., 3D ResNet) extract high-level imaging features, while spatial omics data are encoded via GNNs or CNNs (3). Cross-modal fusion engine, employing three AI architectures—GNN for cross-modal graph alignment, Transformer for attention-based multimodal fusion, and GAN for virtual staining and spatial super-resolution—alongside joint embedding learning (4). Interpretable output and virtual biopsy, generating a comprehensive TIME landscape (e.g., immune cell infiltration, immune checkpoint expression, tertiary lymphoid structures, treatment resistance) with attention heatmaps and XAI techniques (e.g., Grad-CAM) to map macroscopic imaging features to microscopic molecular niches. This framework enables non-invasive, dynamic, and panoramic assessment of the bladder cancer TIME.

Having established a technical bridge mapping macroscopic imaging features to microscopic molecular niches, integrated research on radiomics and spatial omics has progressed from proof-of-concept to concrete clinical applications, with its core output being the gradual realization of a clinically viable “virtual biopsy” paradigm (223, 225, 226). This paradigm aims to systematically and dynamically assess the spatial biological characteristics of the tumor immune microenvironment (TIME) through non-invasive imaging methods, thereby guiding precision diagnosis and treatment (124, 224). The core of the integration of radiomics and spatial omics lies in establishing a quantitative mapping relationship between “imaging features - spatial biology,” thereby enabling non-invasive, panoramic, and dynamic assessment of the tumor immune microenvironment (TIME) (227, 228). Spatial omics provides biological anchors for imaging features. For example, a high tumor-stroma ratio (TSR) predicted by a CT radiomics model corresponds to a microenvironment revealed by spatial omics that is enriched with immunosuppressive cells (such as Tregs) and reduced effector immune cells (229–231). This allows radiomics to transition from a “black box” model to an interpretable biological information carrier. Conversely, radiomics provides a tool for the non-invasive promotion of spatial findings: once spatial omics identifies specific spatial regions (such as tertiary lymphoid structures) with cell or molecular characteristics closely related to prognosis, radiomics can attempt to extract corresponding macroscopic imaging features, thus achieving non-invasive screening and dynamic monitoring of key spatial biological features, marking an important practice towards “virtual biopsy” (232). This integrated paradigm, moving towards a comprehensive “virtual biopsy”, is conceptually summarized in **Figure 3**.

#### 5.2.1 Providing spatial biological interpretation and validation for imaging features

Spatial omics technology can provide a solid molecular and cellular biological foundation for the tumor phenotypes revealed by radiomics, transforming radiomics models from a “black box” into interpretable carriers of biological information. Numerous studies have validated the reliability of this cross-scale association. In bladder cancer, the tumor-stroma ratio (TSR) predicted by a CT-based radiomics model has been confirmed to be closely related to the degree of CD8^+^ T cell infiltration and response to immunotherapy (21, 233). Further spatial biological mechanism studies indicate that “patients with high TSR tumors have lower overall survival and cancer-specific survival rates, and the pathological complete response rate to neoadjuvant chemotherapy significantly decreases. High TSR tumors exhibit a stronger immunosuppressive microenvironment, enriched in regulatory T cells and tumor-associated neutrophils; whereas low TSR tumors are enriched in CD8^+^ effector T cells and dendritic cells” (234, 235). This not only validates the accuracy of radiomics predictions but also endows it with a clear spatial distribution connotation of immune cells.

Similarly, the radiomics assessment of tumor-infiltrating lymphocytes (TILs) is also corroborated through spatial analysis. The Rad-TIL model based on CT imaging shows high discrimination ability (AUCs of 0.844 and 0.816, respectively) and can significantly associate with patients’ survival outcomes and responses to immunotherapy (236). Spatial proteomics analysis reveals that these imaging features actually reflect the spatial patterns of “T cell distributions as ‘rejecting,’ ‘infiltrating,’ or ‘desert’ types” within the tumor, providing important evidence for understanding tumor heterogeneity and formulating individualized treatment strategies (237, 238).

#### 5.2.2 The realization of “Virtual Biopsy” and multimodal data fusion

Supported by the biological foundation provided by spatial omics, the concept of “virtual biopsy” in radiomics and its dynamic monitoring capabilities are gradually expanding in application among solid tumors such as bladder cancer. For instance, researchers successfully predicted the PD-L1 expression status in bladder cancer based on CT radiomic features, providing a non-invasive reference for patient stratification in immunotherapy (239). This type of imaging-based non-invasive classification embodies the core functionality of “virtual biopsy.”

Furthermore, the fusion of multi-sequence MRI radiomics and spatial transcriptomics data is gradually becoming a cutting-edge strategy for reconstructing the spatial map of the tumor microenvironment. Through this fusion strategy, “virtual biopsy” can upgrade from inferring single molecular biomarkers to systematically depicting complex spatial biological maps. Specifically, by extracting spatial heterogeneity features from multi-timepoint, multi-sequence MRI images and spatially registering and integrating them with spatial transcriptomics data, researchers can achieve spatial localization and dynamic tracking of molecular expression patterns in different regions within the tumor (233, 240). In a breast cancer study, a multimodal model was developed by integrating longitudinal MRI spatial habitat radiomics, transcriptomics, and single-cell RNA sequencing data. This model effectively captured dynamic spatial heterogeneity changes in tumors during neoadjuvant therapy. It demonstrated high predictive accuracy (AUC up to 0.888) in both external validation and multi-omics cohorts, and further revealed a link between imaging spatial features and enhanced immune activity, such as B cell infiltration (241). This case provides a feasible technical framework for achieving similar panoramic, dynamic “virtual biopsy” in bladder cancer.

#### 5.2.3 Dynamic monitoring and efficacy evaluation

Another significant advantage of radiomics is its non-invasiveness, allowing for repeated execution during treatment, thus possessing powerful dynamic monitoring capabilities. “Additionally, radiomics can be used for dynamic monitoring of tumor heterogeneity and changes in efficacy, capturing the evolution of the tumor microenvironment and morphology before and after treatment through ‘delta-radiomics’ analysis of imaging features, thereby evaluating efficacy or early identifying trends of resistance” (242). Such dynamic monitoring not only provides new means for precise tumor treatment and follow-up management but also offers data support for exploring the mechanisms of tumor biological evolution.

By correlating such dynamic imaging data with molecular evolution maps obtained through spatial omics before and after treatment, a dynamic and causally meaningful connection can be established between macroscopic imaging changes and microscopic molecular mechanisms, thereby deepening the understanding of treatment responses and resistance mechanisms.

### 5.3 Advantages, limitations, and clinical readiness of radiomics and spatial omics

Radiomics and spatial omics provide complementary yet fundamentally distinct perspectives for deciphering the complexity of the TIME in bladder cancer from macroscopic and microscopic dimensions, respectively. To more clearly evaluate their respective values and challenges, this section systematically compares the advantages, limitations, and clinical readiness of both approaches, aiming to offer a comprehensive perspective for TIME research in bladder cancer.

### 5.3.1 Advantages, limitations, and clinical readiness of radiomics

The core strengths of radiomics lie in its non-invasiveness and holistic nature. It enables high-throughput extraction of macroscopic heterogeneity features from the entire tumor using conventional imaging modalities such as CT and MRI, facilitating a “virtual biopsy” of the tumor’s immune status, and possesses the unique capability for repeated dynamic monitoring throughout the treatment course (243). However, a significant limitation is that radiomic features often lack direct biological interpretability, making it difficult to establish clear causal links with the molecular mechanisms of TIME (244). For instance, while a texture feature may correlate with immune cell infiltration, the underlying biological significance (e.g., reflecting cell density, nuclear-to-cytoplasmic ratio, or collagen fiber arrangement) often remains elusive. Secondly, variations in image acquisition parameters (such as slice thickness, contrast agent dosage, and reconstruction algorithms) and hardware equipment can cause substantial fluctuations in feature values, leading to poor reproducibility of validation results across centers and scanners (245). This technical heterogeneity severely hinders the generalizability of radiomics models. Finally, the diversity in feature selection methods (e.g., LASSO, random forest) and modeling algorithms results in inconsistent study outcomes, accompanied by a lack of standardized analytical workflows and reporting criteria (246). Although guidelines such as the Image Biomarker Standardisation Initiative (IBSI) exist, their implementation in actual research is often not rigorous, making it difficult to directly compare and integrate results across different studies, thereby limiting the potential of radiomics as a reliable tool for clinical decision support.

Furthermore, radiomics has fundamental limitations in resolving the micro-details of the tumor immune microenvironment (TIME), stemming from inherent bottlenecks in imaging resolution. First, radiomics cannot distinguish specific subtypes of immune cells; for instance, it fails to differentiate between CD8+ T cells with anti-tumor effects and regulatory T cells (Tregs) with immunosuppressive functions, nor can it resolve spatial interactions and functional states between cells (247). Such cell-level information is crucial for understanding immune escape mechanisms and predicting responses to immunotherapy. Second, in TIME regions with low cell density or diffuse infiltration, radiomic features may be obscured by the high signals from the tumor bulk, leading to the loss of critical information. For example, in stroma-rich tumor areas, signals from immune cells may be overwhelmed by those from abundant fibroblasts and collagen. Finally, the resolution of conventional imaging (millimeter-scale) is far lower than the cellular scale (micrometer-scale); consequently, it cannot capture microscopic events such as the formation of immune synapses, concentration gradients of cytokines, or nanoscale structural changes in the extracellular matrix (248). These microscopic events are core components of the immune response, and their absence renders radiomics inherently deficient in elucidating the fine-tuned regulatory mechanisms of the TIME, making it difficult to provide depth of information comparable to spatial transcriptomics or multiplex immunofluorescence techniques.

In terms of clinical readiness, radiomics has reached a relatively mature stage and has demonstrated preliminary validation potential in the clinical application of bladder cancer, particularly in using CT imaging to predict muscle-invasive status and lymph node metastasis (249). For instance, studies have constructed models for predicting bladder cancer prognosis by extracting high-throughput features from CT images; these models can quantify intratumoral heterogeneity and provide auxiliary information for clinical decision-making (250). However, the lack of standardized protocols (such as variations in imaging equipment, parameters, and segmentation criteria) and the performance degradation of models during external validation remain major obstacles to their clinical translation. Consequently, radiomics currently serves primarily as an adjunctive tool to supplement traditional radiological assessment and cannot yet replace the gold standard status of pathological biopsy or molecular testing in diagnostic classification. Its true value in supporting clinical decisions, especially in guiding precision therapies such as immunotherapy, still awaits final confirmation through rigorously designed large-scale randomized controlled trials (251).

### 5.3.2 Advantages, limitations, and clinical readiness of spatial omics

Spatial omics (such as spatial transcriptomics and spatial proteomics) provides fine-grained biological resolution at the microscopic scale. The high resolution and capacity to retain spatial information inherent in spatial omics make it a powerful tool for deeply understanding the heterogeneity of the bladder cancer tumor immune microenvironment (TIME) and its immune regulatory mechanisms. Its core advantage lies in its high degree of biological interpretability, directly revealing the spatial localization and functional states of different cell subpopulations (e.g., immune-excluded, infiltrated, or desert-type T cell distributions) (252). However, spatial omics technologies typically rely on high-quality tissue samples, such as fresh-frozen or formalin-fixed paraffin-embedded (FFPE) tissues. Sample quality, particularly RNA integrity, directly impacts data quality; yet, obtaining bladder cancer tissue is often invasive, for instance, via transurethral resection of bladder tumors (TURBT) (253). Secondly, high costs restrict many studies to small-scale explorations, making it difficult to conduct validations with sufficient statistical power. Furthermore, analyzing spatial omics data requires specialized bioinformatics teams, which further raises the threshold and cost of research. Finally, current spatial omics technologies are generally based on tissue sections whose thickness (typically 5–10 micrometers) represents only a two-dimensional cross-section, failing to fully reflect the three-dimensional structure of the TIME. This two-dimensional perspective may lead to an underestimation of spatial heterogeneity; for example, the distribution patterns of tumor-infiltrating lymphocytes in three-dimensional space may differ from those observed in two-dimensional sections (254). Therefore, its invasiveness (reliance on tissue biopsy) and sampling bias constitute fundamental limitations, hindering comprehensive assessment of the entire tumor landscape and precluding dynamic monitoring during treatment. Moreover, spatial omics technologies impose extremely high requirements on sample processing, sequencing depth, and spatial resolution, resulting in high detection costs and long turnaround times, which severely limit their adoption as routine clinical diagnostic tools.

Compared with radiomics, the clinical readiness of spatial omics in the field of bladder cancer lags significantly, currently remaining largely confined to basic and translational research stages. Through methods such as multiplex immunofluorescence or imaging mass cytometry, this technology enables *in situ* characterization of the spatial distribution, phenotypes, and interactions of various immune cells (e.g., T cells, B cells, macrophages) on tissue sections, thereby facilitating the discovery of novel immunotherapy targets or biomarkers (36, 255). Nevertheless, technical bottlenecks impeding its clinical translation are prominent: first, the experimental workflow is protracted, ranging from sample processing and multiple rounds of antibody staining to complex data analysis, potentially taking several weeks and thus failing to meet the need for rapid clinical decision-making; second, the technology entails high costs involving expensive reagents, instruments, and specialized bioinformatics analysis, limiting its widespread application; finally, there is currently a lack of clinically validated, user-friendly automated analysis software, rendering data analysis highly dependent on expert experience and challenging reproducibility (256). Although a few studies have attempted to directly correlate spatial omics findings (such as specific spatial clustering patterns of immune cells) with clinical prognosis, these discoveries cannot yet be directly applied to patient risk stratification and treatment planning due to the absence of standardized quantitative thresholds and independent large-scale validation cohorts (257).

#### 5.3.3 The point of contention between radiomics and spatial omics

The point of contention lies in whether there exists a stable and generalizable mapping relationship between the macroscopic imaging features identified by radiomics and the microscopic molecular niches revealed by spatial omics. For instance, can specific imaging texture patterns consistently correspond to particular immunosuppressive cell communities? Current research has yet to provide a definitive answer, urgently requiring large-scale, prospective multicenter studies to validate these associations and resolve the inconsistencies. Therefore, the most cutting-edge strategy is not to have the two replace each other but to promote AI-driven deep integration. This involves utilizing models like graph neural networks and Transformers to build a quantitative bridge from macroscopic imaging to microscopic molecules, thereby truly achieving the leap from merely “seeing” a tumor to “understanding” it.

### 5.4 Clinical translation: current progress, challenges, and future directions

#### 5.4.1 Current progress and validation needs

Significant progress has been made in the clinical validation and application of radiomics models in bladder cancer (258). Multicenter cohort studies and external validation continuously strengthen the generalization ability of models. For instance, comprehensive prediction tools that integrate clinical variables, such as nomograms, have demonstrated the potential to improve the accuracy of individualized risk assessment. Their predictive performance (e.g., a C-index as high as 0.853) surpasses that of standalone clinical or radiomics models, enabling more precise risk stratification (259, 260). Furthermore, the concepts of “virtual biopsy” and dynamic monitoring are expanding the dimensions of non-invasive tumor characterization (259). In actual clinical scenarios, “the clinical value of virtual biopsy and dynamic monitoring is expected to further enhance with the deep integration of radiomics and multi-omics data, such as spatial transcriptomics and genomics.

However, despite the promising prospects, current studies are mostly single-center retrospective designs with limited sample sizes, making it difficult to fully reflect disease heterogeneity (261, 262). Therefore, promoting large-scale, standardized, prospective multicenter cohort studies is the “gold standard” for validating the clinical value of integrated models and an essential path toward their clinical application (263, 264). Multicenter studies can cover different regions, equipment, and populations, thereby enhancing the external validity and generalizability of models (265). For instance, research has shown that combining MRI radiomics with genomic features can effectively predict CD8A expression, demonstrating high AUC values in both training and validation sets, which suggests potential for cross-center application (266). Only through rigorous, prospective study designs combined with standardized image acquisition and processing workflows can the robustness and usability of radiomics models in different clinical scenarios be ensured (267, 268).

#### 5.4.2 Current technical and clinical challenges

Radiomics and spatial omics have shown great potential in the study of the immune microenvironment of bladder cancer, but there are still many challenges in clinical application. First, the standardization of data acquisition is an urgent issue to be addressed. There are differences in sample collection, processing, and sequencing depth among different centers, platforms, and technical methods, which affect the comparability and reusability of the data. For example, spatial omics has very high requirements for tissue sampling, specimen preservation, and spatial resolution, while radiomics is limited by inconsistencies in imaging equipment, parameter settings, and subsequent image processing workflows (25). These factors collectively impact the stability and generalizability of research findings.

Secondly, the standardization of analysis processes is also a key constraint on technological translation. Currently, the analysis of multi-omics data related to the immune microenvironment of bladder cancer relies heavily on complex bioinformatics workflows, including the identification of immune cell subtypes, screening of immune-related genes, and quantitative analysis of spatial distribution (269, 270). However, the lack of unified software tools and analysis standards not only increases operational difficulty but also makes it challenging to directly compare data between different studies. Some research has attempted to enhance the automation and reproducibility of analysis through algorithm integration and model construction, but further optimization and validation are still needed before large-scale clinical application.

Third, clinical operability and data fusion present significant hurdles. A core challenge lies in effectively fusing highly heterogeneous macroscopic imaging features with microscopic molecular data (271, 272). Simple linear analyses are likely insufficient to capture the complex nonlinear relationships between these modalities. Furthermore, different studies employ vastly different integration strategies (e.g., feature fusion, graph neural networks, generative adversarial networks), and the lack of a unified standard makes it difficult to directly compare and validate results (273).

A related and critical challenge is batch effects when integrating multi-center data to develop robust predictive models. For radiomics, differences in scanner models, scanning parameters (e.g., tube voltage, slice thickness), and reconstruction algorithms across centers lead to shifts in feature distributions. Even with correction methods like ComBat, these differences may not be fully eliminated and could even introduce new spurious associations, thereby affecting model generalizability (274). For example, a study integrating multi-center CT data found that significant differences in feature distributions persisted even after correction, leading to decreased model performance on external validation sets (275). Spatial omics data integration faces similar issues. Variations in sample processing protocols (e.g., tissue fixation time, antibody concentration, staining protocols, sequencing platforms) across laboratories can cause systematic differences in gene expression or protein signal intensity, which can confound downstream biological analyses (276).

This leads to a central controversy in the field: Should model development prioritize:high internal accuracy using high-quality data from a single center, even at the expense of generalizability? Or should it prioritize stronger generalizability through large-scale multi-center data, potentially sacrificing some internal precision? Currently, no consensus exists. Some researchers argue that for clinical decision support tools, generalizability is paramount, thus prioritizing multi-center data. Others caution that batch effects in integrated data may obscure true biological signals, causing models to learn technical artifacts rather than genuine biological associations, and therefore advocate for a more cautious approach to multi-center data.

#### 5.4.3 Future directions

To overcome the aforementioned challenges and advance the field, future efforts should focus on the following directions (1): AI-driven deep Integration: Utilizing advanced AI models such as graph neural networks to naturally handle spatial relationship data, and deeply explore the nonlinear associations between imaging and spatial omics data.This includes employing cross-modal graph contrastive learning, Transformer-based multimodal attention fusion, and Generative Adversarial Networks (GANs) for virtual staining techniques to establish quantitative mapping relationships from macroscopic imaging features to microscopic molecular niches (2). Promoting prospective Multicenter Cohort Studies: This is the “gold standard” for validating the clinical value of integrated models, and a necessary step for their clinical application. Large-sample, multicenter studies can not only verify the stability and generalizability of biomarkers but also reveal the heterogeneity of the immune microenvironment across different populations and bladder cancer subtypes (3). Developing a closed-loop diagnostic verification platform: Combining technologies such as organoids and liquid biopsies to create a precise diagnostic and therapeutic closed loop of “non-invasive assessment → spatial verification → treatment guidance → dynamic monitoring,” accelerating technological iteration.Such a closed-loop platform aims to integrate non-invasive assessment results with histological spatial validation, thereby forming a continuously optimized diagnostic and therapeutic cycle (4). Establishing standardized processes and a data sharing ecosystem: It has become a consensus to establish standardized processes covering sample collection, spatial annotation, data processing, and result interpretation. At the same time, building an open spatial omics and imaging omics database and promoting standardized sharing of research data will help improve data utilization efficiency and facilitate resource integration and collaborative innovation within the field.Promoting data sharing and standardization through the establishment of international collaborative networks will significantly accelerate the clinical translation of related technologies.

In summary, although radiomics and spatial omics have made significant progress in researching the immune microenvironment of bladder cancer, their clinical application still faces multiple challenges, including data standardization, analytical workflows, and operational feasibility. Through multicenter collaboration, optimization of technical workflows, and translational research oriented toward clinical needs, it is expected that this field will move from the laboratory to the clinic, bringing more precise and efficient diagnosis and treatment solutions to bladder cancer patients.

## 6 Conclusion and future perspectives

The high heterogeneity of the immune microenvironment in bladder cancer is a core determinant of its variable clinical behavior and treatment responses. This review systematically discusses how radiomics and spatial omics, from both macro and micro perspectives, provide us with unprecedented insights to unravel this complexity. Radiomics enables non-invasive quantitative assessment of the global heterogeneity of tumors, and its “virtual biopsy” capability is gradually moving towards clinical application; spatial omics, on the other hand, meticulously depicts the cellular geography and interaction networks of the TIME, fundamentally deepening our understanding of immune mechanisms. However, the most revolutionary prospect lies in the deep integration of the two. By combining the macro-navigation capabilities of radiomics with the micro-analytical capabilities of spatial omics, we hope to build a bridge from “seeing” tumors to “understanding” tumors, driving a profound shift in oncology research paradigms from “single molecular biomarkers” to “spatial ecosystems.” Looking ahead, overcoming data and algorithmic challenges and conducting prospective clinical validation will be key to realizing this vision and ultimately providing truly personalized precision treatment for bladder cancer patients.
