# Supplementary material for: Perspective on the integration of radiomics and spatial omics in the analysis of the tumor microenvironment of bladder cancer and prospects for precision diagnosis and treatment
Source: Front Immunol. 2026 Jul 8;17:1821743. doi: 10.3389/fimmu.2026.1821743 (PMC13388900; doi:10.3389/fimmu.2026.1821743)
Supplement: Supplementary file 1 [file Supplementaryfile1.pdf]

Supplemental materials

3.2. Clinical applications of radiomics in precision immunotherapy

The ultimate goal of decoding the TIME is to optimize clinical decision-making. Radiomics demonstrates tremendous translational potential in this regard, providing important tools for the precise implementation of immunotherapy in bladder cancer through non-invasive assessment of the tumor immune microenvironment status.

3.2.1. Prediction of immunotherapy response

Radiomics models have become powerful tools for predicting the efficacy of immune checkpoint inhibitors (ICIs). For example, in high-risk non-muscle invasive bladder cancer, CT radiomic features combined with non-negative matrix factorization (NMF) algorithms can effectively predict the risk of failure of BCG immunotherapy (1). For advanced patients, radiomics models developed based on CT are significantly correlated with ICI efficacy across various solid tumors (including bladder cancer), with AUCs reaching as high as 0.74, and predictive performance improves further when combined with clinical variables (2).

3.2.2. Prognostic assessment and risk stratification

Radiomics also plays a core role in the prognostic assessment of bladder cancer. Radiomic features extracted from multi-sequence MRI are significantly associated with the recurrence-free survival of bladder cancer patients. The radiomics-clinical nomogram model constructed by integrating radiomic features with clinical risk factors has a predictive performance (C-index up to 0.853) that surpasses that of standalone clinical or radiomics models, achieving more precise risk stratification(3). For metastatic risk, a nomogram model based on arterial phase CT radiomic features and pathological T staging demonstrates good efficacy in predicting the risk of metastatic recurrence after radical cystectomy (validation group AUC reaching 0.883)(4).

3.2.3. Guiding individualized treatment strategies

By non-invasively assessing the TIME status, radiomics is gradually becoming an auxiliary tool for guiding individualized treatment decisions. Its ability for "virtual biopsy" can screen for populations likely to benefit from immunotherapy before treatment, avoiding ineffective therapies. Furthermore, its dynamic monitoring capability during treatment helps in the early identification of resistance signs, providing a basis for timely adjustments to treatment plans.

3.3. Challenges and limitations

3.3.1 Data heterogeneity and standardization issues

Despite its promising prospects, bladder cancer radiomics must overcome numerous challenges before becoming a routine clinical application. Data heterogeneity is the primary barrier; differences in imaging devices and scanning parameters among various medical institutions directly impact the stability of features and the generalizability of models. For example, images obtained from magnetic resonance imaging (MRI) and computed tomography (CT) across different manufacturers, models, and parameter settings—even for the same patient and the same location—may exhibit differing gray level distributions, spatial resolutions, and noise levels. Although there are variations in equipment and protocols, the diagnostic accuracy of radiomics models in predicting muscle-invasive bladder cancer has shown relative homogeneity(5).

The absence of standardized processes permeates the entire workflow of image segmentation,

feature extraction, and analysis. Currently, commonly used features in radiomics research include multi-dimensional parameters such as shape, texture, and intensity distribution. However, these features are highly sensitive to the preprocessing methods applied to the raw image data (e.g., resampling, normalization, denoising). The lack of a unified feature extraction and processing workflow can result in entirely different feature sets from the same image across different studies or platforms, subsequently affecting the stability and reproducibility of downstream machine learning models. Systematic reviews indicate that despite methodological differences among studies, the diagnostic accuracy of radiomics models in predicting muscle-invasive bladder cancer is relatively balanced, suggesting that some features may possess a higher cross-platform stability(5). This highlights that standardizing feature extraction and analysis workflows can enhance the reproducibility of models and lay the groundwork for multi-center collaboration and large-scale clinical promotion. In response to data heterogeneity and standardization issues, several quality assessment and process standardization tools have been proposed internationally, such as the Radiomic Quality Score (RQS) and the Quality Assessment of Diagnostic Accuracy Studies-2 (QUADAS-2), to evaluate bias risks in study design and data processing. These tools help unify quality standards in radiomics research and promote the establishment of common data collection and analysis norms within the field. In the future, as multi-center large sample collaborative research advances, standardized radiomics databases and sharing platforms will further facilitate external validation and clinical translation of models.

In summary, data heterogeneity and standardization issues are core challenges that urgently need to be addressed in current bladder cancer radiomics research. Through unifying equipment parameters, standardizing feature extraction processes, and promoting high-quality research standards, it is hoped that the stability and generalizability of radiomics models can be improved, providing more reliable technical support for the precise assessment and individualized management of the bladder cancer immune microenvironment.

3.3.2. Barriers and strategies for clinical translation applications

The clinical translation applications of radiomics models currently face numerous obstacles. Despite the promising potential of radiomics and deep learning models based on CT, MRI, etc., for assessing the immune microenvironment in bladder cancer and predicting responses to immunotherapy, their promotion in real clinical environments still encounters many challenges.

Firstly, the generalization ability of the models is limited by the heterogeneity of training data, including differences in imaging devices, scanning parameters, tumor segmentation standards, and patient populations. Secondly, the extraction and interpretation of radiomics features have not yet been standardized, leading to significant reproducibility differences between studies. Additionally, the performance of models in external validation cohorts is often lower than that in internal validations, indicating that their stability and transferability need further improvement. Considering these issues, only through rigorous multi-center, prospective research designs, combined with standardized imaging acquisition and processing workflows, can the robustness and usability of radiomics models be ensured across different clinical scenarios. It is noteworthy that joint modeling of radiomics and clinical variables can enhance predictive capability, providing new ideas for the clinical translation of models(6).

Moreover, existing studies are mostly single-center retrospective designs with limited sample sizes, making it difficult to comprehensively reflect disease heterogeneity. Therefore, promoting large-scale, standardized, multi-center prospective research that integrates multi-omics data and

establishes unified analytical processes is a key strategy for improving model robustness and facilitating the clinical translation of radiomics. On one hand, multi-center studies can encompass diverse regions, different devices, and varied populations, enhancing the generalization ability and external verifiability of the models. For example, MRI radiomics combined with genomic features can effectively predict CD8A expression, showing high AUC values in both training and validation groups, indicating its potential for cross-center applicability(7). On the other hand, prospective studies can reduce selection bias and information bias, ensuring the actual effectiveness of models in dynamic clinical processes. Furthermore, with the rise of multi-omics technologies such as single-cell and spatial omics, future multi-center studies need to integrate multidimensional data to deeply reveal the complexity of the tumor immune microenvironment, thereby providing more reliable bases for personalized immunotherapy decisions (8). Currently, there is a lack of large-scale, standardized, prospective multi-center cohort studies, which has become one of the main bottlenecks for the clinical translation of radiomics models. Establishing an international collaboration network to promote data sharing and standardization would greatly accelerate the clinical implementation of related technologies.

Overall, radiomics provides us with a powerful non-invasive assessment tool by transforming conventional medical imaging into high-dimensional data that reflects the tumor immune microenvironment. It can macro-decode immune cell infiltration, stromal ratios, and key molecular expressions, thereby demonstrating tremendous value in predicting immunotherapy responses, prognostic assessments, and personalized treatments. With the advancement of standardization processes and the deep integration of artificial intelligence technologies, radiomics is expected to become an important bridge connecting macro imaging and the micro immune environment, promoting the development of precision medicine in bladder cancer. However, to deeply interpret the specific cellular interactions and molecular pathways behind these macro imaging features, we will need to utilize the spatial omics technologies, which will be elaborated in the next chapter, capable of precise localization at the microscopic scale.

4.3. Application of spatial genomics in bladder cancer immunotherapy

4.3.1 Spatial biomarkers for predicting immunotherapy response

Spatial genomics technologies, particularly spatial transcriptomics and spatial proteomics, provide unprecedented resolution for revealing key spatial biomarkers that predict the efficacy of immune checkpoint inhibitors (ICI) in the immune microenvironment of bladder cancer. Recent studies have found that the spatial distribution of the tumor stem-like cell (CD274+ALDH+) subpopulation in muscle-invasive bladder cancer is closely related to immune suppression status and patient survival rates. Multi-omics analyses show that IGF2BP3 and SPHK1 are upregulated in these cells and are closely associated with poor response to immunotherapy and prognosis. Further insights from spatial transcriptomics and CODEX multiplex protein detection reveal that IGF2BP3+SPHK1+ tumor cells are spatially closer to exhausted CD8+ T cells, providing a basis for explaining why some patients respond better to immunotherapy. An ICI-related score based on the gene characteristics enriched in IGF2BP3+SPHK1+ tumor cells has been validated for its predictive capability in independent immunotherapy cohorts, reinforcing its clinical application prospects as a spatial biomarker(9). Notably, spatial genomics also reveals the impact of tumor regional heterogeneity on predicting immunotherapy response, suggesting that spatial biomarkers may offer greater accuracy than traditional overall molecular markers.

The association between the spatial distribution of immune cells and treatment sensitivity has also received strong support from spatial omics. Analysis of protein marker expression in different regions of bladder cancer tissue through digital spatial proteomics (DSP) revealed that markers such as PD-L1, Ki-67, and HLA-DR are closely related to pathological responses in specific spatial areas (such as tumor-enriched regions, immune-enriched regions, and tumor-immune interfaces). Notably, high expression of HLA-DR in the tumor-immune interface region is positively correlated with good treatment responses. The accuracy of predicting immunotherapy responses (AUC=0.827) significantly improved when machine learning models integrated spatial protein markers compared to models without spatial partitioning, indicating that spatial distribution features have significant added value in predicting immunotherapy responses(10). Based on this, spatial omics can also reveal changes in the distance between immune cells and tumor cells in the tumor microenvironment, such as increased spatial separation between immune cells and tumor cells after chemotherapy, which may lead to suppression of anti-tumor immune responses, thereby affecting the sensitivity to immunotherapy(11). These pieces of evidence suggest that close spatial contact between immune cells and tumor cells may be a key premise for benefiting from immunotherapy.

Moreover, spatial omics has also propelled the discovery of novel molecular markers. For instance, the spatial expression of UCK2 in bladder cancer primarily concentrates on malignant tumor cells and stromal cells and is associated with the expression of immune checkpoint molecules, immune cell recruitment, and activation. Patients with high UCK2 expression showed a higher response rate during PD-L1 inhibitor treatment, indicating that UCK2 is not only a driving factor for tumor progression and metabolic reprogramming but also a potential biomarker for predicting immunotherapy responses on a spatial level(12). Similarly, the spatial heterogeneity of CD73 expression has been confirmed to be closely related to the tumor immune microenvironment and prognosis, with high levels of CD73+ regulatory T cell infiltration in tumor tissues considered an independent adverse prognostic factor, potentially influencing the spatial regulatory effects of the PD-1/PD-L1 pathway(13). These findings collectively indicate that spatial omics can not only reveal the spatial distribution patterns of traditional molecular markers but also discover new spatial biomarkers, providing more precise evidence for personalized decision-making in immunotherapy.

In summary, spatial omics technology is driving the prediction of immunotherapy responses in bladder cancer from static molecular markers to dynamic spatial biomarkers. In the future, with the further integration of spatial multi-omics and artificial intelligence technologies, spatial biomarkers are expected to achieve routine clinical application, providing a solid foundation for precise stratification and efficacy enhancement of immunotherapy for bladder cancer patients.

4.3.2 Spatial analysis of mechanisms of immune treatment resistance

The occurrence of immune treatment resistance in bladder cancer is closely related to the spatial dynamics of various cell populations within the tumor microenvironment. Spatial omics technologies, especially spatial transcriptomics and digital spatial analysis, can reveal the spatial distribution of different cell types within tumor tissue and their transcriptional features associated with resistance. For instance, spatial omics analysis of bladder cancer samples before and after treatment with immune checkpoint inhibitors (CPI) found that resistant subtypes (such as Luminal-excluded type) exhibited specific genetic and transcriptomic programs(14). In non-muscle invasive bladder cancer, comparative analysis of spatial molecular characteristics shows significant differences in expression profiles between treatment responders and non-responders, where the

tumor regions of non-responders often lack dynamic changes in immune response gene expression, suggesting that a spatially "inactive" or "cold" immune microenvironment is an important basis for resistance(15).

The spatial dynamic changes of drug-resistant related cell populations are not limited to tumor cells themselves but also involve immunosuppressive cells and stromal components. Single-cell and spatial transcriptomic co-analysis shows that immunosuppressive cells such as cancer-associated fibroblasts are enriched at the tumor margin or specific subregions, forming an immune barrier that hinders the infiltration and activation of effector T cells(16). In bladder cancer, cells with high expression of LRFN2 within the tumor reduce the recruitment and functional transformation of CD8+ T cells by inhibiting the secretion of pro-inflammatory cytokines, thereby spatially forming an "exclusion" relationship with CD8+ T cells, which is particularly pronounced in resistant patients(17). Furthermore, FOS+ B cells, as newly discovered immune resistance-related subpopulations, have been shown to be closely associated with poor responses to immunotherapy across various tumors, with their spatial distribution highly overlapping with tumor immunosuppressive regions, indicating their critical role in shaping the resistant microenvironment(18).

Spatial omics demonstrate unique advantages in elucidating resistance mechanisms. Firstly, spatial omics can simultaneously capture information on cell types, molecular expression, and spatial location at the tissue in situ level, revealing the heterogeneity and dynamic evolution of the tumor microenvironment. For example, spatial transcriptomics has revealed a spatially specific enhancement of lactic acid metabolic reprogramming in the regions enriched with tumor cells, which is closely related to resistance to immune checkpoint inhibitors(19). In bladder cancer, the spatial heterogeneity of taurine metabolic dysregulation affects not only tumor cells but also reshapes the distribution and functions of fibroblasts and macrophages, promoting the formation of an immunosuppressive microenvironment and the occurrence of immune therapy resistance(20). Spatial omics can also reveal tumor subclones and their evolutionary trajectories, helping to identify clonal expansions and spatial ecological niche changes associated with resistance (21). These findings suggest that the spatial relationships and signaling interactions between tumor cells, immune cells, and stromal cells are one of the core mechanisms of immune resistance.

Furthermore, spatial omics technology can provide a basis for molecular typing of resistance mechanisms and patient stratification. Through spatial transcriptomic analysis, it is possible to identify molecular features and spatial biomarkers associated with resistance, laying the groundwork for accurately predicting responses to immunotherapy and developing novel combination treatment strategies(22). For instance, spatial omics analysis of patients with high TMs (taurine metabolic dysregulation index) reveals that their tumor regions are enriched with immunosuppressive cell populations, express high levels of immune checkpoint molecules, and activate Notch and EGFR resistance pathways, providing theoretical support for targeting metabolic pathways in combination with immunotherapy(20). With the integration of spatial multi-omics with artificial intelligence and big data analysis, there is hope for a panoramic and dynamic resolution of the immune resistance mechanisms in bladder cancer, promoting advances in personalized precision treatment.

In summary, spatial omics technology significantly expands our understanding of the immune resistance mechanisms in bladder cancer immunotherapy. By revealing the spatial dynamic changes

of resistance-related cell populations, analyzing the complex structure of the tumor microenvironment, and its molecular regulatory network, it provides a solid foundation for molecular typing of resistance mechanisms, patient stratification, and the discovery of new therapeutic targets. The continued development of spatial omics will help tackle the clinical challenge of immune resistance, advancing the practice of precision immunotherapy for bladder cancer.

4.3.3 Design of individualized immunotherapy strategies

Spatial omics technology provides a solid foundation for the fine analysis of the immune microenvironment in bladder cancer and the design of individualized immunotherapy strategies. Through systematic analysis of the spatial distribution, gene expression, and functional status of different cell types within tumor tissues, researchers can accurately identify cell subpopulations, metabolic pathways, and key molecules closely related to immune responses. For instance, large-scale single-cell RNA sequencing and spatial transcriptomics data reveal that the high expression of glycolysis-related genes (such as COPB2) is closely associated with an immunosuppressive microenvironment and poorer immunotherapy prognosis; Glycolysis.Sig may serve as a novel biomarker for predicting immunotherapy response(23). By integrating spatial omics information, it is possible to not only screen for patient subpopulations sensitive to immunotherapy but also discover new therapeutic targets such as glycolytic pathways, immune-related genes, chemokines, and their receptors, thereby providing theoretical basis and data support for individualized immunotherapy.

Spatial information can also guide the optimization of combined treatment plans. The heterogeneity of the tumor microenvironment in bladder cancer limits the effectiveness of single immunotherapy regimens; spatial omics analysis can reveal the distribution characteristics of immunosuppressive cells, tumor-associated fibroblasts, and metabolic abnormalities in different regions of the tumor. For example, spatial omics combined with deep learning image analysis can non-invasively and quantitatively assess tumor microenvironment heterogeneity (such as the tumor-stroma ratio, TSR) and its relationship with CD8+ T cell infiltration and responses to immunotherapy, assisting clinicians in formulating more precise combination treatment strategies(6). In addition, spatial omics technologies can also be used to screen molecular targets that synergize with therapies such as immune checkpoint inhibitors, chemotherapy, and radiotherapy, achieving the optimal combination of immunotherapy and traditional treatments.

In terms of individualized model construction, the immune-related gene pair (IRGP) model developed based on RNA-seq and microarray data can effectively predict the prognosis and immunotherapy response of bladder cancer patients(24). These models, combined with spatial distribution information, are expected to overcome the shortcomings of traditional molecular typing that neglects spatial heterogeneity, providing more personalized treatment recommendations for patients. It is noteworthy that spatial omics has also promoted the establishment of in vitro personalized drug sensitivity models, such as tumor organoids rapidly constructed using acoustic droplet printing technology, which can retain the in situ immune microenvironment and be used to assess the interactions between autologous immune cells and tumor cells, providing a high-throughput, reproducible platform for screening and efficacy prediction of personalized immunotherapy regimens(24). The combination of spatial omics with emerging technologies such as organoids and co-culture with immune cells can further enhance the precision and practicality of

personalized treatments.

In summary, spatial omics technology provides a solid theoretical and technical foundation for the personalization and precision of immunotherapy in bladder cancer by revealing the spatial heterogeneity of the bladder cancer immune microenvironment, screening precise immunotherapy targets, optimizing combination treatment strategies, and promoting the establishment of personalized models and drug sensitivity platforms. In the future, with the deep integration of spatial omics with multi-omics and artificial intelligence technologies, personalized immunotherapy strategies are expected to achieve higher levels of precision matching and efficacy improvement.

References

1. Ye L, Chen Y, Xu H, Wang Z, Li H, Qi J, et al. Radiomics of Contrast-Enhanced Computed Tomography: A Potential Biomarker for Pretreatment Prediction of the Response to Bacillus Calmette-Guerin Immunotherapy in Non-Muscle-Invasive Bladder Cancer. Front Cell Dev Biol. 2022;10:814388.
2. Ligero M, Garcia-Ruiz A, Viaplana C, Villacampa G, Raciti MV, Landa J, et al. A CT-based Radiomics Signature Is Associated with Response to Immune Checkpoint Inhibitors in Advanced Solid Tumors. Radiology. 2021;299(1):109-19.
3. Yang G, Bai J, Hao M, Zhang L, Fan Z, Wang X. Enhancing recurrence risk prediction for bladder cancer using multi-sequence MRI radiomics. Insights Imaging. 2024;15(1):88.
4. Lv H, Zhou X, Liu Y, Liu Y, Chen Z. Feasibility analysis of arterial CT radiomics model to predict the risk of local and metastatic recurrence after radical cystectomy for bladder cancer. Discov Oncol. 2024;15(1):40.
5. Kozikowski M, Suarez-Ibarrola R, Osiecki R, Bilski K, Gratzke C, Shariat SF, et al. Role of Radiomics in the Prediction of Muscle-invasive Bladder Cancer: A Systematic Review and Meta-analysis. Eur Urol Focus. 2022;8(3):728-38.
6. Du Y, Sui Y, Tao Y, Cao J, Jiang X, Yu J, et al. Comprehensive Assessment of Tumor Stromal Heterogeneity in Bladder Cancer by Deep Learning and Habitat Radiomics. Acad Radiol. 2025;32(12):7244-59.
7. Zheng Z, Guo Y, Huang X, Liu J, Wang R, Qiu X, et al. CD8A as a Prognostic and Immunotherapy Predictive Biomarker Can Be Evaluated by MRI Radiomics Features in Bladder Cancer. Cancers (Basel). 2022;14(19).
8. Grausenburger R, Herek P, Shariat SF, Englinger B. Recent contributions of single-cell and spatial profiling to the understanding of bladder cancer. Curr Opin Urol. 2024;34(4):236-43.
9. Wang Y, Song W, Feng C, Wu S, Qin Z, Liu T, et al. Multi-omics analysis unveils the predictive value of IGF2BP3/SPHK1 signaling in cancer stem cells for prognosis and immunotherapeutic response in muscle-invasive bladder cancer. J Transl Med. 2024;22(1):900.
10. Beckabir W, Wobker SE, Damrauer JS, Midkiff B, De la Cruz G, Makarov V, et al. Spatial Relationships in the Tumor Microenvironment Demonstrate Association with Pathologic Response to Neoadjuvant Chemoimmunotherapy in Muscle-invasive Bladder Cancer. Eur Urol. 2024;85(3):242-53.
11. Chelushkin MA, van Dorp J, van Wilpe S, Seignette IM, Mellema JJ, Alkemade M, et al. Platinum-Based Chemotherapy Induces Opposing Effects on Immunotherapy Response-Related

Spatial and Stromal Biomarkers in the Bladder Cancer Microenvironment. Clin Cancer Res. 2024;30(18):4227-39.
12. Guo Y, Lin Z, Zhang W, Chen H, Chen Y, Liu Y, et al. Comprehensive multi-omics analysis of nucleotide metabolism: elucidating the role and prognostic significance of UCK2 in bladder cancer. Funct Integr Genomics. 2025;25(1):133.
13. Izawa M, Tanaka N, Murakami T, Anno T, Teranishi Y, Takamatsu K, et al. Single-Cell Phenotyping of CD73 Expression Reveals the Diversity of the Tumor Immune Microenvironment and Reflects the Prognosis of Bladder Cancer. Lab Invest. 2023;103(4):100040.
14. Robertson AG, Meghani K, Cooley LF, McLaughlin KA, Fall LA, Yu Y, et al. Expression-based subtypes define pathologic response to neoadjuvant immune-checkpoint inhibitors in muscle-invasive bladder cancer. Nat Commun. 2023;14(1):2126.
15. Meghani K, Frydenlund N, Yu Y, Choy B, Meeks JJ. A Spatial Comparison of Molecular Features Associated with Resistance to Pembrolizumab in BCG Unresponsive Bladder Cancer. medRxiv. 2023.
16. Li Y, Huan C, Sun H, Zhang W, Guo Z, Li C, et al. Spatial Transcriptomics and snRNA-seq Expose CAF Niches Orchestrating Dual Stromal-Immune Barriers in Hepatocellular Carcinoma. Adv Sci (Weinh). 2025:e14661.
17. Yu A, Hu J, Fu L, Huang G, Deng D, Zhang M, et al. Bladder cancer intrinsic LRFN2 drives anticancer immunotherapy resistance by attenuating CD8(+) T cell infiltration and functional transition. J Immunother Cancer. 2023;11(10).
18. Zhang X, Ma J, Chen Y, Deng X, Zhang Y, Han Y, et al. FOS(+) B cells: Key mediators of immunotherapy resistance in diverse cancer types. Mol Ther Oncol. 2024;32(4):200895.
19. Fu S, Xu J, Wang C, Zhang C, Li C, Xie W, et al. Cancer specific up-regulated lactate genes associated with immunotherapy resistance in a pan-cancer analysis. Heliyon. 2024;10(23):e39491.
20. Liang Z, Nong F, Li Z, Chen R, Zhao H, Huang Y. Taurine-mediated metabolic immune crosstalk indicates and promotes immunosuppression with anti-PD-1 resistance in bladder cancer. Front Immunol. 2025;16:1618439.
21. Zhang Y, Wang W. Advances in tumor subclone formation and mechanisms of growth and invasion. J Transl Med. 2025;23(1):461.
22. Liu D, Xiao L, Wu Y, Yue C, Li M, Sun Y, et al. Spatially Resolved Transcriptomics: Revealing Tumor Microenvironment Heterogeneity to Advance Cancer Immunotherapy. Small Methods. 2025;9(11):e00770.
23. Li Y, Yang W, Chen H, Jin Z, Dong J, Ma L, et al. Comprehensive pan-cancer single-cell analysis reveals glycolysis-related signatures as predictive biomarkers for immunotherapy response and their role in bladder cancer. Int Immunopharmacol. 2025;152:114381.
24. Zhang LH, Li LQ, Zhan YH, Zhu ZW, Zhang XP. Identification of an IRGP Signature to Predict Prognosis and Immunotherapeutic Efficiency in Bladder Cancer. Front Mol Biosci. 2021;8:607090.
